# Conception and Synthesis of Sequence‐Coded Morpholinos

**DOI:** 10.1002/chem.202501161

**Published:** 2025-04-29

**Authors:** Benoit Pousse, Abdelaziz Al Ouahabi, Paul N. W. Baxter, Laurence Charles, Jean‐François Lutz

**Affiliations:** ^1^ CNRS ISIS Université de Strasbourg 8 allée Gaspard Monge Strasbourg 67000 France; ^2^ CNRS Institut Charles Sadron UPR22 Université de Strasbourg 23 rue du Loess Cedex 2 Strasbourg 67034 France; ^3^ CNRS UMR 7273 Institute of Radical Chemistry Aix Marseille Université Cedex 20 Marseille 13397 France

**Keywords:** digital polymers, phosphorus chemistry, sequence‐controlled polymers, sequencing, solid‐phase synthesis

## Abstract

Solid‐phase morpholino chemistry was explored as a new route to synthesize abiological sequence‐defined oligomers. Two comonomers, 0 and 1 containing (i) a chlorophosphoramidate reactive function, (ii) a trityl‐protected morpholine, and (iii) a coding substituent (H or CH_3_ for 0 and 1, respectively) on the morpholine ring were first synthesized and characterized. This binary alphabet was afterwards tested for the synthesis of digitally‐encoded oligomers with different lengths and sequences. The oligomers were prepared on a modified polystyrene resin, cleaved, and characterized by liquid chromatography mass spectrometry. When using a repetitive cycle containing only morpholino coupling and trityl deprotection steps, the formed oligomers were not uniform. Thus, an additional capping step was added. In these conditions, uniform coded sequences were prepared in most cases. Furthermore, the oligomers were analyzed by tandem mass spectrometry. In the studied collision‐induced dissociation conditions, the repeat units of the oligomers undergo two main‐chain fragmentations and full sequence coverage was observed for all studied sequences. Therefore, the binary messages stored in the oligomers could be decoded and retrieved in all cases.

## Graphical abstract

Morpholino oligomer chemistry, which relies on the coupling of a chlorophosphoramidate group with the secondary amine of a morpholine ring, has been to date only explored for the synthesis of xeno nucleic acids. Here, we report that it can also be used for the design of digital polymers. Oligomers with different sizes and sequences were synthesized using a binary monomer alphabet.

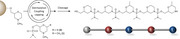

## 1 Introduction

In recent years, manmade sequence‐defined polymers (SDPs) have emerged as a new class of functional macromolecules.^[^ ^1^ ^]^ Like biopolymers such as proteins and nucleic acids, they contain perfectly‐defined monomer sequences.^[^ ^1b^ ^]^ Originally, most of these synthetic polymers have been conceived as biological mimics (i.e., peptidomimetics,^[^ ^2^ ^]^ xeno nucleic acids^[^ ^3^ ^]^) and used in bio‐applications, for example as antisense, biocides, artificial enzymes, or inhibitors.^[^ ^4^ ^]^ More recently, a new generation of abiotic SDPs has been developed and applied in materials science,^[^ ^5^ ^]^ for example for data storage, anti‐counterfeiting technologies, and plastic recycling.^[^ ^6^ ^]^ Since the molecular design of these polymers is not limited by biological considerations, various types of chemistries have been reported for their preparation.^[^ ^7^ ^]^

Yet, the synthesis of SDPs remains tedious.^[^ ^5d^ ^]^ Indeed, these polymers are prepared by multistep syntheses, which are often slow and limited in yields.^[^ ^8^ ^]^ Currently, the most efficient approach for the preparation of SPDs is phosphoramidite polymer chemistry (PPC).^[^ ^9^ ^]^ For instance, it allows synthesis of long polymer chains containing more than hundred residues.^[^ ^10^ ^]^ Although originally developed for oligonucleotide synthesis,^[^ ^11^ ^]^ this P(III) chemistry was afterwards applied to the synthesis of other types of polymers including digital polymers,^[^ ^12^ ^]^ foldamers,^[^ ^13^ ^]^ and polymer bioconjugates.^[^ ^14^ ^]^ In general, phosphoramidite chemistry is more robust than P(V) strategies that have initially been investigated for the chemical synthesis of nucleic acids.^[^ ^15^ ^]^ Yet, the relevance of P(V) chemistry for oligonucleotide synthesis has recently been rehabilitated by Baran and coworkers in a series of elegant publications.^[^ ^16^ ^]^ Among P(V)‐mediated reactions, the coupling of chlorophosphoramidate with the secondary amine of a morpholine ring has already been proven suitable for oligomer synthesis.^[^ ^17^ ^]^ Initially reported by Summerton and Weller,^[^ ^18^ ^]^ the repetition of this coupling step leads to the formation of phosphorodiamidate morpholino oligomers (PMO, also commonly named morpholinos), which have been mainly explored as antisense agents (i.e. oligomers that can bind a specific DNA sequence).^[^ ^19^ ^]^ Thus, to date, original morpholino coupling, as well as more recent alternative strategies,^[^ ^20^ ^]^ have only been tested with nucleobase‐containing monomers. It was therefore tempting to explore this chemistry for the preparation of other types of precision oligomers such as digital macromolecules.^[^ ^6b^ ^]^ In this context, we describe herein the preparation of digitally‐encoded morpholinos. Two coded monomers that do not contain a nucleobase substituent were conceived and tested for the solid‐phase synthesis of oligomers.

## 2 Results and Discussion

**Scheme 1:**
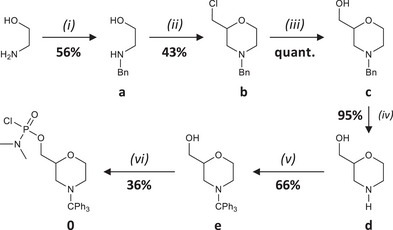
Synthesis of monomer **0**. Experimental conditions: (i) benzaldehyde, Na_2_SO_4_, MeOH, rt, overnight, then NaBH_4_, 0 °C, 3 h; (ii) epichlorhydrin, rt, overnight then H_2_SO_4_, 120 °C, 2 h; (iii) formamide, H_2_O, 145 °C, overnight; (iv) Pd/C, H_2_, MeOH, rt, 6 days; (v) Ph_3_C‐Cl, Et_3_N, DCM, rt, overnight; (vi) LiBr, DBU, POCl_2_NMe_2_, DCM, 0 °C, 15 min.

Solid‐phase PMO synthesis requires monomers that contain a chlorophosphoramidate reactive group and a trityl‐protected morpholine. In the present study, a binary alphabet based on two coded monomers, **0** and **1**, which do not contain any nucleobase, was designed. Monomer **0** was synthesized in six steps (Scheme 1). The synthesis was started with the protection of ethanolamine through a reductive amination (step (i) in Scheme 1) to afford **a**. Afterwards, the morpholine ring of **b** was formed by reacting **a** with epichlorhydrin under harsh conditions (step (ii) in Scheme 1). Intermediate **c** was obtained in quantitative yield by keeping **b** at 145 °C overnight under reflux in the presence of water and formamide (step (iii) in Scheme 1). No column chromatography was required after this step. The protecting benzyl group was afterwards removed using Pd/C under H_2_ atmosphere (step (iv) in Scheme 1) to afford **d**,^[^ ^21^ ^]^ which was then tritylated into **e** (step (v) in Scheme 1).^[^ ^22^ ^]^ To avoid compound degradation after this step, purification has to be performed using deactivated silica and triethylamine. Finally, monomer **0** was obtained by coupling the alcohol of **e** with dichlorophosphoramidate reagent (step (vi) in Scheme 1). Due to poor separation and potential degradation, the purification of **0** required two successive columns on deactivated silica (see Experimental section for details).

**Scheme 2:**
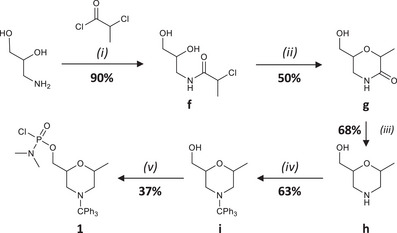
Synthesis of monomer **1**. Experimental conditions: (i) Et_3_N, ACN, MeOH, ‐10 °C, overnight; (ii) tBuOK, tAmOH, rt, 3 h; (iii) LiAlH_4_, THF, 0 °C, 2 days; (iv) Ph_3_C‐Cl, Et_3_N, DCM, 0 °C, overnight; (v) POCl_2_NMe_2_, DBU, LiBr, DCM, 0 °C, 15 min.

Monomer **1** was prepared in 5 steps (Scheme 2). First, intermediate **f** was obtained in very good yields by reacting aminopropanediol with chloropropionyl chloride (step (i) in Scheme 2). Afterwards, **f** was cyclized into 6‐(hydroxymethyl)‐2‐methylmorpholin‐3‐one **g** (step (ii) in Scheme 2), which was then reduced into **h** using LiAlH_4_ (step (iii) in Scheme 2). The final two steps of this synthesis were the same as for the synthesis of **0**. Intermediate **i** was first obtained by tritylation of **h** (step (iv) in Scheme 2). Ultimately, monomer **1** was obtained by reacting the alcohol group of **i** with dichlorophosphoramidate reagent (step (v) in Scheme 2).

**Scheme 3:**
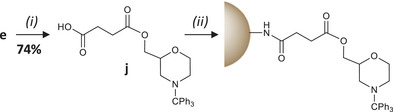
Modification of the polystyrene solid‐support used in this work. Experimental conditions: (i) succinic anhydride, DMAP, pyridine, 0 °C, 2 h; (ii) aminomethyl polystyrene, DMAP, DCC, DCM, rt, overnight.

A modified polystyrene resin was also prepared (Scheme 3). Although controlled pore glass is usually the best solid support for automated synthesis of nucleic acids and xeno nucleic acids,^[^ ^23^ ^]^ polystyrene resins are more practical for explorative manual synthesis.^[^ ^12^, ^24^ ^]^ First, the reactive spacer **j** containing a carboxylic acid group was formed by reacting succinic anhydride with **e** (step (i) in Scheme 3). The excess of succinic anhydride must be fully removed after this step in order to ensure an efficient resin modification. The spacer **j** was then reacted with a commercial aminomethyl polystyrene resin (step (ii) in Scheme 3), thus affording a preloaded solid‐support containing a tritylated morpholine ring.

**Scheme 4:**
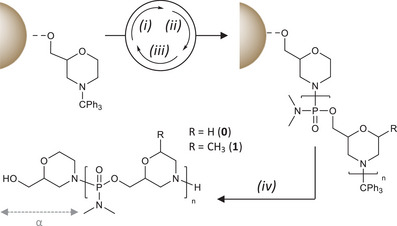
General strategy for the solid‐phase synthesis of sequence‐coded PMOs. Experimental conditions: (i) 20% TFA in DCM (3×10 min + 2×5 min); (ii) **0** or **1** (4 eq), ETT (10 eq), NEM (10 eq), NMP; (iii) Ac_2_O, NMP (20/80) and NMI, NMP (40/80); (iv) NH_4_OH, 60–65 °C, overnight.

The morpholino oligomers were synthesized via a multistep growth polymerization mechanism.^[^ ^8^ ^]^ It relies on a repetitive cycle involving three successive reactions (Scheme 4). First, the trityl group is removed under acidic conditions (step (i) in Scheme 4). The free secondary amine is then coupled with **0** or **1** (step (ii) in Scheme 4). For the synthesis of some oligomers, a capping step was also used to trap unreactive amines (step (iii) in Scheme 4). The cycle was repeated a certain number of times in order to attain a desired chain length. Ultimately, the formed oligomers are cleaved from the solid support (step (iv) in Scheme 4).

Table 1 lists all the sequences that were synthesized and characterized in this work. Initially, some oligomers were synthesized without a capping step (i.e., using only steps (i) and (ii) of Scheme 4 in the repetitive cycle). In this case, the oligomers were detrytilated on the resin then cleaved. However, the formed PMO were not uniform. The coupling yields were not excellent, probably because of the use of the polystyrene resin. Therefore, a capping step using acetic anhydride was implemented in the repetitive cycle. In this case, the targeted sequences terminated by a trityl group (i.e., trityl‐on strategy) were first cleaved from the resin and then separated from shorter acetylated chains by reverse‐phase column purification. The conditions commonly used for reverse‐phase chromatography of phosphate‐containing polymers were not suitable for PMO and were adapted for optimal purification (see Experimental section). Ultimately, the oligomers were detritylated and analyzed.

**Table 1:** Digital morpholinos that were prepared in this work.

	Sequence^[^ ^a^ ^]^	Mass [Da]	*m/z*_calc_	*m/z*_exp_^[^ ^b^ ^]^
P1	α0	323.1610	324.1683	324.1684^[^ ^c^ ^]^
P2	α1	337.1767	338.1839	338.1842^[^ ^c^ ^]^
P3	α00	529.2430	530.2503	530.2503^[^ ^c^ ^]^
P4	α11	557.2743	558.2816	558.2815^[^ ^c^ ^]^
P5	α01	543.2587	544.2660	554.2658^[^ ^c^ ^]^
P6	α000	735.3251	736.3223	736.3328^[^ ^c^ ^]^
P7	α110	763.3564	764.3636	764.3636^[^ ^c^ ^]^
P8	α0101	969.4384	970.4457	970.4465^[^ ^c^ ^]^
P9	α000000	1353.5712	677.7929	677.7953^[^ ^d^ ^]^

^[a]^ The Greek letter α describes the morpholin‐2‐ylmethanol end‐group coming from the solid‐support, as shown in Scheme 4.

^[b]^ Measured by positive mode electrospray ionization high‐resolution mass spectrometry (ESI‐HRMS).

^[c]^ Measured as [M+H]^+^.

^[d]^ Measured as [M+2H]^2+^.

**Figure 1:**
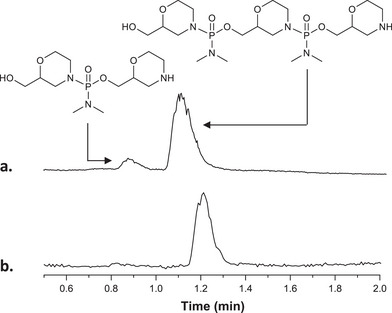
LC measurements recorded for oligomer **P3** synthesized using either (**a**) no capping steps and therefore no purification by reverse‐phase chromatography (**b**) acetic anhydride capping steps and purification by reverse‐phase chromatography.

Figure 1 compares liquid‐chromatography (LC) measurements obtained for oligomer **P3** synthesized with or without capping steps. In the absence of capping steps, a non‐uniform sample is obtained. Indeed, the targeted oligomer α00 co‐exists with a small fraction of shorter chains with sequence α0. This result was confirmed by direct introduction ESI‐HRMS analysis, which also reveals the presence of shorter chains (Figure S1a). Yet, when capping steps were used, uniform samples were detected by both LC (Figure 1b) and ESI‐HR‐MS (Figure S1b). Therefore, capping conditions were applied for the synthesis of all oligomers listed in Table 1. In all cases, the targeted species were obtained, as evidenced by HR‐MS measurements (Figures S2–S9). Despite the use of capping steps, the spectra of the longest sequences (Figures S8,S9) also contained shorter defect sequences.

**Figure 2:**
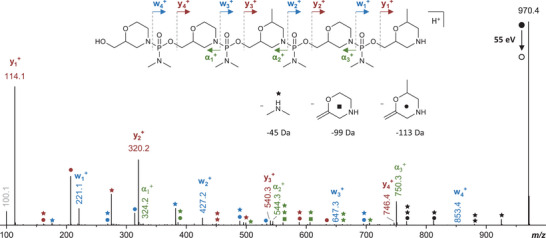
MS/MS spectrum (55 eV laboratory frame) of oligomer **P8** obtained by collision‐induced dissociation of the [**P8**+H]^+^ precursor ion. Symbols designate fragments obtained after loss of dimethylamine (45 Da, star), 2‐methylenemorpholine (99 Da, square), or 2‐methylene‐6‐methylmorpholine (113 Da, circle) and combination thereof from the precursor ion (black) or from fragments α^+^ (green), w^+^ (blue), and y^+^ (red). Other internal fragments are in grey. For the sake of clarity, only the most abundant signals have been annotated, and their complete list can be found in Tables S1–S4.

Nevertheless, the presence of truncated species did not prevent all the oligomers displayed in Table 1 to be efficiently sequenced by MS/MS as such experiments include selection of precursor ions based on their *m/z* value prior to their activation. However, dissociation rules had first to be established for the investigated species as their fragmentation behavior in the positive ion mode strongly differs from PMOs that include nucleobase‐containing monomers. Indeed, the dominant fragmentation pathway observed during collision‐induced dissociation (CID) of protonated PMOs is production of protonated bases.^[^ ^25^ ^]^ This evidences that the positive charges are located on the bases, which prevents any backbone bond cleavages useful for sequencing. Absence of nucleobase in the digital morpholinos oligomers studied here offers an alternative protonation scenario and so alternative CID pathways. Figure 2 shows the MS/MS spectrum obtained for the **P8** oligomer. When subjected to CID, three main fragment series were formed upon cleavage of backbone bonds. Protonation of the N atom in any morpholine ring induces the cleavage of the N─P bond, producing w_j_^+^ ions as proposed in Scheme S1a. Alternatively, transfer of the proton from the CH group of one morpholine ring to the N atom of the preceding ring induces the loss of one (CH_3_)_2_N─PO_2_ molecule and generates either α^+^ or y^+^ fragments depending on the location of the adducted H^+^ (Scheme S1b). Two main additional reactions are also observed to occur in a successive manner: loss of dimethylamine (45 Da, indicated by stars in Figure 2) and, as depicted in Scheme S1c, elimination of the last morpholine ring as a neutral which mass depends on this last coding unit, that is, 99 Da for **0** (designated by squares) or 113 Da for **1** (designated by circles). These secondary reactions occur from both the [P+H]^+^ precursor ion and all fragments (accurately mass measured in Tables S1‐S4). They could be minimized by proper adjustment of the collision energy, yet at the expense of an abundance of useful product ions, notably the smallest members of the α_n_^+^ series. Indeed, using optimal CID conditions, these sequencing fragments are of low abundance but still perfectly detected, as supported by the quality of accurate mass measurements reported in Table S2). For example, the weakest member of this series, α_0_^+^ at *m/z* 118.2 (not annotated in Figure 2 for the sake of clarity), has a relative abundance of only 0.2% but a signal‐to‐noise ratio (S/N) of 120, enabling high accuracy measurement with relative error of −0.8 ppm. Actually, the occurrence of secondary reactions raises other issues: not only they increase the complexity of MS/MS spectra but they also generate product ions that can interfere with w^+^ and y^+^ sequencing fragments. For example, the w_1_^+^ ion is expected at *m/z* 207.1 or *m/z* 221.1 depending on the last coding unit being 0 or 1, respectively: in Figure 2, peaks are observed at these two *m/z* values and prior knowledge of the **P8** sequence is required to properly assign w_1_^+^ at *m/z* 221.1 and the *m/z* 207.1 fragment to the loss of a 113.1 neutral from y_2_^+^ at *m/z* 320.2. Similarly, the signal at *m/z* 100.1 (or *m/z* 114.1) can either be identified as y_1_^+^ containing the last **0** (or **1**) coding unit or the protonated form of the 99.1 Da (or 113.1 Da) neutral. In contrast, unambiguous sequencing is always achieved with α^+^ fragments. The first member of this series, α_0_^+^, does not contain any coding unit: even when it is no longer detected as the oligomer length increases, its *m/z* 118.1 value can be used as the starting point to reconstruct the oligomer sequence by searching fragments after iterative addition of 206 Da (for **0**) or 220 Da (for **1**) up to the precursor ion. In Figure 2, α_1_^+^ is found at *m/z* 324.2 (= 118.1 + 206) and there is no signal at *m/z* 338.2 (= 118.1 + 220), so the first coding unit is 0. Similarly, unique values are found for α_2_^+^ (*m/z* 544.3 = α_1_^+^ + 220) and α_3_^+^ (*m/z* 750.3 = α_2_^+^ + 206) while [**P8**+H]^+^ at *m/z* 970.4 (= α_3_^+^ + 220) reveals the last increment. Accordingly, the 0101 sequence is determined for **P8**, which now permits to accurately assign w^+^ and y^+^ series for the sake of validation. The same procedure also applies for multiply charged precursor ions preferentially produced as the oligomer length increases, which permitted full sequence coverage of all oligomers in this study (Figures S10–S15). Due to the aforementioned sequencing rules, these digital oligomers are not the easiest to read, notably compared to other species such as oligourethanes.^[^ ^26^ ^]^ Yet, in contrast to most other families developed by our group, their full sequence coverage can be achieved from protonated molecules which permits to capitalize on the renown high ionization yield of positive mode ESI to ensure sufficient S/N for all sequencing fragments. Therefore, sequence‐encoded PMOs constitute an interesting new family of polymers for digital storage.

## 3 Conclusion

In summary, morpholino chemistry was investigated herein for the first time for the synthesis of non‐antisense oligomers; for instance, for the design of digitally‐encoded molecules. Two monomers of different molar mass were conceived and synthesized in order to prepare a binary alphabet that can be deciphered by tandem mass spectrometry sequencing. Monomer **0** containing no substituent on the 6‐position of the morpholine ring was prepared in six steps, whereas monomer **1** containing a 6‐methylated morpholine core was obtained in five steps. This alphabet allowed solid‐phase synthesis of digitally‐encoded PMOs with different chain‐length and monomer sequences. Yet, an acetic anhydride capping step was implemented in order to reduce the fraction of truncated sequences. The formed digital PMOs were analyzed by MS/MS and could be efficiently decoded.

This first proof‐of‐principle indicates that the coupling of chlorophosphoramidate with the secondary amine of a morpholine ring is a valid chemistry for the preparation of non‐natural informational oligomers. In the present study, a morpholine ring was kept in the molecular design because of prior art in the antisense field.^[^ ^17^ ^]^ However, the main role of morpholine rings in PMOs is to mimic ribose rings found in nucleic acids. This is important for xeno nucleic acids design but maybe not for the preparation of digital polymers. Hence, in future studies, the coupling of chlorophosphoramidate with non‐cyclic secondary amines may also be considered. Additionally, as mentioned in this article, the use of polystyrene resin is practical for exploratory studies but probably not optimal for the synthesis of longer sequence‐defined chains. Therefore, the chemistry reported herein may also be applied to other types of supports (e.g., controlled pore glass) and eventually automatized.

## Conflict of Interests

The authors declare no conflict of interest.

## Supporting information



Supporting Information

## Data Availability

The data that support the findings of this study are available from the corresponding author upon reasonable request.
