# Supplementary material for: Conception and Synthesis of Sequence‐Coded Morpholinos
Source: Chemistry. 2025 Apr 29;31(29):e202501161. doi: 10.1002/chem.202501161 (PMC12099184; doi:10.1002/chem.202501161)
Supplement: Supplementary file 1 — Supporting Information [file CHEM-31-e202501161-s001.pdf]

Supporting Information for

Conception and synthesis of sequence-coded morpholinos

Benoit Pousse,[a, b] Abdelaziz Al Ouahabi,[b] Paul N. W. Baxter,[a, b] Laurence Charles,*[c] and Jean-François Lutz*[a, b]

[a] Université de Strasbourg, CNRS, ISIS, 8 allée Gaspard Monge, 67000 Strasbourg, France
E-mail: jflutz@unistra.fr

[b] Université de Strasbourg, CNRS, Institut Charles Sadron UPR22 23 rue du Loess, 67034 Strasbourg Cedex 2, France

[c] Aix Marseille Université, CNRS, UMR 7273, Institute of Radical Chemistry, 13397, Marseille Cedex 20, France
E-mail: laurence.charles@univ-amu.fr

Table of contents:

A. Experimental procedures S2
A.1. Materials and reagents S2
A.2. Synthesis of monomer **0** S3
A.3. Synthesis of monomer **1** S6
A.4. Resin modification S9
A.5. Solid-phase synthesis of PMOs S10

B. Measurements and analyses S11
B.1. Nuclear magnetic resonance S11
B.3. Mass spectrometry S11

C. Additional data and figures S12

D. References S36

A. Experimental procedures

A.1. Materials and reagents. Ethanolamine (98%, Alpha Aesar), benzaldehyde (>98%, TCI), sodium borohydride ($NaBH_4$, 99%, Sigma Aldrich), epichlorohydrin (>99%, TCI), palladium on carbon (Pd/C, 10 wt.% loading, Sigma Aldrich), chlorotriphenylmethane (98%, ABCR), lithium bromide (LiBr, ≥99%, Sigma Aldrich), 1,8-diazabicyclo[5.4.0]undec-7-ene (DBU, 98%, TCI), chloropropionyl chloride (97%, Sigma Aldrich), aminopropanediol (3-amino-1,2-propanediol, 99%, Sigma Aldrich), lithium aluminum hydride (1.0 M in THF, Sigma-Aldrich), 4-(dimethylamino)pyridine (DMAP, 99%, Alpha Aesar), succinic anhydride (≥99%, Sigma Aldrich), copper sulfate ($CuSO_4$, ≥99%, Sigma Aldrich), dicyclohexylcarbodiimide (DCC, 99%, Alpha Aesar), N,N-diisopropylmethylphosphonamidic chloride ($POCl_2NMe_2$, 95%, ABCR), anhydrous sodium sulfate (Na_2SO_4, ≥98%, VWR), anhydrous magnesium sulfate (MgSO4, >97%, VWR), sulfuric acid (95-97%, Sigma Aldrich), sodium hydroxide (>98%, Fisher), potassium sodium tartrate tetrahydrate (Rochel salt, 99%, Sigma Aldrich), triethylamine (Et_3N, 98%, Thermo Scientific), potassium tert-butoxide (tBuOK, 97%, Thermo Scientific), N,N-diisopropylethylamine (DIPEA, 99%, TCI), acetic anhydride (Ac_2O, ≥99%, Sigma Aldrich), N-methylimidazole (NMI, 99%, Alpha Aesar), 4-ethylmorpholine (NEM, 98%, Alpha Aesar), trifluoro acetic acid (TFA, ≥99.98%, Roth), methanol (MeOH, 99.9%, Carlo Erba), ethyl acetate (EtOAc, 99.8%, Carlo Erba), toluene (99.5% Carlo Erba), cyclohexane (pur, Carlo Erba), formamide (≥99.0% Sigma Aldrich), anhydrous methanol (99.9%, Sigma Aldrich), anhydrous dichloromethane (DCM, >99.8% stabilized with 50 ppm amylene, Sigma Aldrich), dichloromethane (DCM, 99.8%, Carlo Erba), acetonitrile (HPLC grade ≥99.9%, Sigma Aldrich), 2-methyl-2-butanol (tAmOH, 99%, Sigma Aldrich), anhydrous tetrahydrofuran (THF, >99.9% inhibitor-free, Sigma Aldrich), anhydrous pyridine (99.8% in Sure/Seal™, Sigma Aldrich), pyridine (≥99.5%, Sigma Aldrich), 1-methyl-2-pyrrolidinone (NMP, 99%, Alpha Aesar), aqueous ammonia (28% in water, VWR), silica gel for chromatography column (high-purity grade, pore size 60 Å, 230 - 400 mesh, SiO_2, VWR), sodium chloride (for brine solution, Cedo), Celite 545, aminomethylated polystyrene HL (100-200 mesh, (loading: 1.25 mmol/g, Sigma Aldrich), activation reagent (0.25 M 5-(ethylthio)-1H-tetrazole in MeCN, ETT, ChemGenes), deactivated silica (500 mL of silica with 50 mL of water), Glen-pak DNA purification cartridge (10 nmole - 1.0 µmole, Glen Research).

A.2. Synthesis of monomer 0. The 6-steps synthetic route for preparing monomer **0** is shown in Scheme 1 of the main document. ·Corresponding NMR spectra are shown in Figures S16-S27.

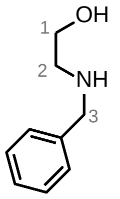

Chemical Formula: $C_9H_{13}NO$
Molecular Weight: 151.21 $g{\cdot}mol^{-1}$

Synthesis of intermediate a. To a solution of ethanolamine (10 mL, 1.5 equiv, 0.167 mol) in MeOH (200 mL), Na_2SO_4 (32 g, 2.04 equiv, 0.225 mol), and benzaldehyde (11.77 mL, 1 equiv, 0.113 mol) were added. The mixture was stirred overnight at room temperature. $NaBH_4$ (6.32 g, 1.5 equiv, 0.167 mol) was added at 0°C and stirred for 3 hours. Subsequently, 50 mL of water was added to quench the reaction, and the mixture was stirred for an additional 10 minutes. The mixture was then filtered to remove the maximum amount of salt, and the aqueous phase was extracted with EtOAc (3 x 100 mL). The combined organic layers were washed with water (100 mL), brine (100 mL), and then dried over $MgSO_4$ yielding the product (14.3602 g, 0.095 mol, 56%) as a yellow oil. ^1H NMR (400 MHz, $CDCl_3$) δ 7.40-7.21 (m, 5H, H_{Ar}), 3.79 (s, 2H, H_3) 3.64 (m, 2H, H_2) 2.78 (m, 2H, H_1).^{13}C NMR (101 MHz, $CDCl_3$) 140.09, 128.80, 128.49, 127.46, 61.12 (C_2), 53.78 (C_3), 50.85 (C_1). HRMS (*m/z*): calculated for $C_9H_{14}NO^+$ $[M+H]^+$: 152.1070, found: 152.1068

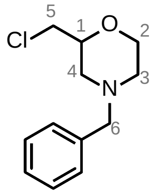

Chemical Formula: $C_{12}H_{16}ClNO$
Molecular Weight: 225.72 $g{\cdot}mol^{-1}$

Synthesis of intermediate b. 2-(benzylamino)ethan-1-ol **a** (14.3602 g, 1 equiv, 0.095 mol)) was stirred with epichlorohydrin (8.19 mL, 1.1 equiv, 0.104 mol) at room temperature overnight. Then, H_2SO_4 (95%) (18 mL, 3.54 equiv, 0.336 mol) was added and the mixture was stirred at 120°C for 2 hours. A 40% solution of NaOH was added to reach pH=14. The aqueous phase was extracted with toluene (3 x 100 mL). The combined organic layers were washed with brine (2 x 100 mL) and then dried over $MgSO_4$. The crude was purified by column chromatography (SiO_2, 800 mL, 20 cm, EtOAc/Cyclohexane 0.5/9.5 to 5/5) yielding the product (9.0987 g, 43%, 0.040 mol) as orange oil. ^1H NMR (400 MHz, $CDCl_3$) δ 7.34 – 7.16 (m, 5H, H_{Ar}), 3.85 (ddd, J = 11.3, 3.3, 2.0 Hz, 1H, H_2), 3.74 – 3.68 (m, 1H, H_1), 3.65 (td, J = 11.3, 2.6 Hz, 1H, $H_{2'}$), 3.58 – 3.38 (m, 4H, 2 x H_6 + H_5 + $H_{5'}$), 2.78 (dt, J = 11.1, 2.0 Hz, 1H, H_4), 2.60 (dq, J = 11.5, 2.1 Hz, 1H, H_3), 2.15 (td, J = 11.3, 3.3 Hz, 1H, $H_{3'}$), 1.95 (dd, J = 11.2, 9.7 Hz, 1H, $H_{4'}$). ^{13}C NMR (101 MHz, $CDCl_3$) δ 137.86, 129.45, 128.67, 127.59, 75.49 (C_1), 67.20 (C_2), 63.51 (C_6), 56.20 (C_4), 53.07 (C_3), 45.29 (C_5). HRMS (*m/z*): calculated for $C_{12}H_{17}NOCl^+$ $[M+H]^+$: 226.0993, found: 226.0991

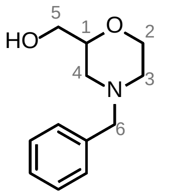

Chemical Formula: $C_{12}H_{17}NO_2$
Molecular Weight: 207.27 $g \cdot mol^{-1}$

Synthesis of intermediate c. 4-benzyl-2-(chloromethyl)-morpholine **b** (9.0987 g, 1 equiv, 0.040 mol) was stirred in 130 mL of formamide/water (100/30: v/v) mixture at 145°C overnight. After the completion of the reaction, 50 mL of water was added, followed by a 40% solution of NaOH to reach pH=14. The aqueous phase was extracted with toluene (4 x 100 mL). The combined organic layers were washed with water (50 mL), brine (50 mL) and dried over $MgSO_4$, resulting in a quantitative yield as a yellow pale oil (8.35 g, 0.040 mol). ^{1}H NMR (500 MHz, $CDCl_3$) δ 7.33 – 7.17 (m, 5H, H_{Ar}), 3.84 (ddd, J = 11.4, 3.3, 1.9 Hz, 1H, H_2), 3.71 – 3.39 (m, 6H, $H_{2'}$+ H_1 + 2 x H_5+ 2 x H_6), 2.63 (m, 2H, H_4 + H_3), 2.13 (td, J = 11.4, 3.4 Hz, 1H, $H_{3'}$), 1.95 (dd, J = 11.3, 10.0 Hz, 1H, $H_{4'}$). ^{13}C NMR (126 MHz, $CDCl_3$) δ 137.81, 129.53, 128.64, 127.57, 76.30 (C_1), 66.95 (C_2), 64.57 (C_5), 63.68 (C_6), 54.88 (C_4), 53.38 (C_3). HRMS (*m/z*): calculated for $C_{12}H_{18}NO_2^+$ $[M+H]^+$: 208.1332, found: 208.1328

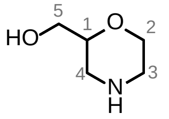

Chemical Formula: $C_5H_{11}NO_2$
Molecular Weight: 117.15 $g \cdot mol^{-1}$

Synthesis of intermediate d. This step was adapted from the literature.[1] Pd/C 10% (1.0334 g, 14% w/w) was added to a dry three neck round bottom flask under argon atmosphere and 70 mL of dry methanol was added. In another flask (4-benzylmorpholin-2-yl)methanol **c** (7.0664 g, 1 equiv, 0.034 mol) was dissolved in 10 mL of anhydrous methanol and added to the first flask. The mixture was flushed several times with H_2. After one day, 0.549 g of Pd/C were added and the mixture was flushed several times with H_2. After three days, 0.527 g of Pd/C were added and the mixture was flushed several times with H_2. After three more days the reaction was completed. The crude product was filtrated over a celite pad washed with DCM (450 mL of DCM was used for this filtration) yielding the product (3.8112 g, 95%, 0.0325 mol) as a pale yellow. The product was pure enough to be used without further purification. ^{1}H NMR (500 MHz, $CDCl_3$) δ 3.92 – 3.87 (m, 1H, H_2), 3.73 – 3.44 (m, 4H, H_1 + H_5 + $H_{2'}$ + $H_{5'}$), 2.94 – 2.76 (m, 3H, H_3 + H_3 + H_4), 2.73 – 2.61 (m, 1H, $H_{4'}$). ^{13}C NMR (126 MHz, $CDCl_3$) δ 77.36 (C_1), 68.18 (C_2), 64.44 (C_5), 47.77 (C_4), 46.15 (C_3). HRMS (*m/z*): calculated for $C_5H_{11}NO_2^+$ $[M+H]^+$:118.0863, found: 118.0865

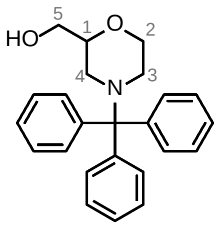

Chemical Formula: $C_{24}H_{25}NO_2$
Molecular Weight: 359.47 g·mol^{-1}

Synthesis of intermediate e. This step was adapted from the literature.[2] To a solution of morpholin-2-ylmethanol **d** (3.65 g, 1 equiv, 0.0311 mol) in 15 mL of anhydrous DCM at 0°C was added triethylamine (5.5 mL, 1.1 equiv, 0.0342 mol). In another flask, (9.67 g, 1.1 equiv, 0.0342 mol) chlorotriphenylmethane was dissolved in 20 mL of anhydrous DCM and this solution was added slowly to the first flask. The mixture was stirred overnight at room temperature. After completion of the reaction, the solvent was removed under vacuum and the crude diluted with 100 ml of ethyl acetate. The organic phase was washed with water (1 x 100 mL), brine (1 x 100 mL), and dried over $MgSO_4$. The crude was purified by column chromatography (deactivated SiO_2, 400 mL, 20 cm, EtOAc/Cyclohexane 2/8 + 2% Et_3N), yielding the product (7.39 g, 66 %,0.0205 mol) as white powder. To avoid broad signals in ^{1}H NMR analysis, the temperature of the spectrometer was cooled at 253 K . ^{1}H NMR (400 MHz, $CDCl_3$) δ 7.53-7.47 (m, 6H, H_{Ar}), 7.34 – 7.24 (m, 6H, H_{Ar}), 7.20-7.13 (m, 3H, H_{Ar}), 4.04 – 3.91 (m, 2H, H_1 + H_2), 3.88 (m, 1H, $H_{2'}$), 3.59 – 3.43 (m, 2H, H_5 + $H_{5'}$), 2.94 (m, 2H, H_3 + H_4), 1.99 (s, 1H, OH), 1.71 (t, J = 11.8 Hz, 1H, $H_{3'}$), 1.49 (t, J = 10.7 Hz, 1H, $H_{4'}$). ^{13}C NMR (101 MHz, $CDCl_3$) δ 129.71, 127.96, 126.53, 77.32 (C_1), 67.55 (C_2), 64.59 (C_5), 50.02 (C_4), 48.48 (C_3). HRMS (*m/z*): calculated for $C_{24}H_{25}NO_2Na^+$ $[M+Na]^+$: 382.1778, found: 382.1773

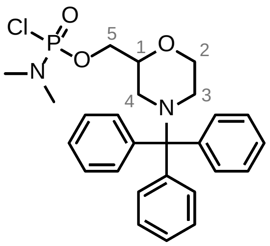

Chemical Formula: $C_{26}H_{30}ClN_2O_3P$
Molecular Weight: 484.96 g·mol^{-1}

Synthesis of 0. (4-tritylmorpholin-2-yl)methanol **e** (1.4123 g, 1 equiv, 3.93x10^{-3} mol) and LiBr (1.6965 g, 4 equiv, 1.57x10^{-2} mol) were added to a dry flask under argon. 20 mL of dry DCM was added and the flask was cooled at 0°C. DBU (2.4 mL, 4 equiv, 1.57x10^{-2} mol). After 5 minutes, $POCl_2NMe_2$ (0.52 mL, 1.1 equiv, 3.93x10^{-3} mol) was added dropwise. After 15 min and the completion of the reaction, the mixture was filtered through a cotton and the solvent was remove. Two columns were done to purify properly the product (SiO_2 deactivated 100 mL, 7 cm, DCM as eluent and SiO_2 deactivated 400 mL, 22 cm, DCM as eluent) yielding the product (689.3 mg, 36 %, 1.42x10^{-3} mol) as a white powder. To avoid broad signals in ^{1}H NMR analysis, the temperature of the spectrometer was cooled at 253°K. Peaks of the two diastereomers are overlapped, only one diastereomer described. ^{1}H NMR (500 MHz, $CDCl_3$) δ

7.39 – 7.13 (m, 15H), 4.15 – 3.82 (m, 5H, H_1, H_2, H_5), 3.04 – 2.90 (m, 2H, H_3, H_4), 2.63 (d, *J* = 13.9 Hz, 6H, NMe_2), 1.63 (m, 1H, $H_{3'}$), 1.47 – 1.31 (m, 1H, $H_{4'}$). ^{13}C NMR (101 MHz, $CDCl_3$) δ 146.90, 129.36, 127.94, 127.67, 127.26, 126.28, 74.25 (C_1), 67.99 (C_2), 67.13 (C_5), 49.84 (C_4), 48.05 (C_3), 36.68 ($N(CH_3)_2$), 36.65 ($N(CH_3)_2$). HRMS (*m/z*): calculated for $C_{26}H_{30}N_2O_3PClNa^+$ $[M+Na]^+$: 507.1575, found: 507.1578

A.3. Synthesis of monomer 1. The 5-steps synthetic route for preparing monomer **1** is shown in Scheme 2 of the main document. The synthesis of intermediates **f**, **g** and **h** was adapted from the literature.[3] Corresponding NMR spectra are shown in Figures S28-S37.

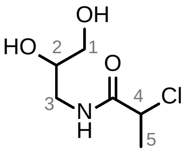

Chemical Formula: $C_6H_{12}ClNO_3$
Molecular Weight: 181.62 g·mol^{-1}

Synthesis of intermediate f. Aminopropanediol (477 mg, 1 equiv, 5.23 mmol) was dissolved in a 20 mL of MeOH/MeCN (3/17; v/v) mixture and cooled to -10 °C using an ice-salt bath. Subsequently, Et_3N (0.85 mL, 1.2 equiv, 6.28 mmol) was added dropwise rapidly. After a 5-minute interval, chloropropionyl chloride (0.81 mL, 1.1 equiv, 5.753 mmol) was introduced dropwise at a rate of 5 mL/h. The resulting mixture was stirred overnight. Solvents were removed under vacuum and the crude was directly purified via column chromatography (SiO_2, 150 mL, 15 cm, MeOH/EtOAc 5/95 to 1/9), yielding the product (863.2 mg, 90%, 4.75 mmol) as a colorless viscous oil. Peaks of the two diastereomers described are overlapped. ^{1}H NMR (400 MHz, $CDCl_3$) δ 4.45 (q, *J* = 7.2 Hz, 1H, H_{4a}), 4.45 (q, *J* = 7.1 Hz, 1H, H_{4b}), 3.83 (m, 2H, H_{2a} + H_{2b}), 3.67 – 3.32 (m, 8H, 2 x H_{1a} + 2 x H_{1b} + 2 x H_{3a} + 2 x H_{3b}), 1.75 (d, *J* = 7.1 Hz, 3H, H_{5a}), 1.75 (d, *J* = 7.1 Hz, 3H, H_{5b}). ^{13}C NMR (101 MHz, $CDCl_3$) δ 171.72 (C=O_a+ C=O_b), 71.10 ($C2_a$+ $C2_b$), 64.08 ($C1_a$+ $C1_b$), 56.00 ($C4_a$+ $C4_b$), 42.74 ($C3_a$+ $C3_b$), 22.89 ($C5_a$+ $C5_b$). HRMS (*m/z*): calculated for $C_6H_{13}NO_3Cl^+$ $[M+H]^+$: 182.0578, found: 182.0578

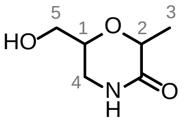

Chemical Formula: $C_6H_{11}NO_3$
Molecular Weight: 145.16 g·mol^{-1}

Synthesis of intermediate g. To a solution of tBuOK (8.801 g, 3 equiv, 78 mmol) in 130 mL of tAmOH, a solution of 2-chloro-N-(2,3-dihydroxypropyl)propanamide **f** (5.6985 g, 1 equiv, 31.4 mmol) in 100 mL of tAmOH was added dropwise during 1h. After completion of the reaction, the mixture was filtered through a silica pad set with EtOAc. After the deposit of

the crude, the pad was rinsed with 500 mL of a mixture 9/1 EtOAc/MeOH. Then, the raw compound was purified by column chromatography (SiO_2, 400 mL, 20 cm, EtOAc/MeOH 98/2 to 9/1) yielding the product (2.6834 g, 50%, 156.2 mmol) as a sticky oil. The two diastereomers described are not present in the same proportion (b>a). ^{1}H NMR (400 MHz, $CDCl_3$) δ 4.42 (q, *J* = 7.1 Hz, 1H, H_{2a}), 4.24 (d, *J* = 6.8 Hz, 1H, H_{2b}), 4.03-3.96 (m, 1H, H_{1a}), 4.00-3.84 (m, 1H, H_{1b}), 3.78 – 3.60 (m, 4H, H_{4a} + $H_{4a'}$ + H_{4b} + $H_{4b'}$), 3.48 – 3.20 (m, 4H, H_{5a} + $H_{5a'}$ + H_{5b} + $H_{5b'}$), 1.47 (d, *J* = 7.1 Hz, 3H, H_{3a}), 1.47 (d, *J* = 6.9 Hz, 3H, H_{3b}). ^{13}C NMR (101 MHz, $CDCl_3$) δ 172.74 (C=O_a), 172.38 (C=O_b), 74.30 (C_{1a}), 73.64 (C_{1b}), 72.05 (C_{2a}), 67.92 (C_{2b}), 63.27 ($C_{4a/b}$), 63.02 ($C_{4a/b}$), 43.64 ($C_{5a/b}$), 43.38 ($C_{5a/b}$), 17.79 ($C_{3a/b}$), 17.56 ($C_{3a/b}$). HRMS (*m/z*): calculated for $C_6H_{12}NO_3^+$ $[M+H]^+$: 146.0812 found: 146.0810

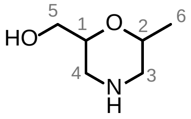

Chemical Formula: $C_6H_{13}NO_2$
Molecular Weight: 131.18 g·mol^{-1}

Synthesis of intermediate h. To a solution of 6-(hydroxymethyl)-2-methylmorpholin-3-one **g** (0.9116 g, 1 equiv, 6.28 mmol) in 20 mL of dry THF at 0 °C, $LiAlH_4$ (1M in toluene, 27 mL, 4 equiv) was added dropwise to the reaction mixture. After one night, 2 equiv of reducing agent was added again. After 40 h, the reaction was quench by adding Rochel salt (saturated solution, 20 mL) and mix with 100 mL of DCM for 2 h. The mixture was filtered through a celite pad washed with DCM (250 mL). No further purification was done yielding the product (68%, 0.5662 g, 4.31 mmol) as a yellow pale oil. The two diastereomers described are not present in the same proportion (a>b). ^{1}H NMR (500 MHz, $CDCl_3$) δ 4.07 (pd, *J* = 6.5, 3.1 Hz, 1H, H_{2b}), 3.98 – 3.85 (m, 1H, H_{5b}), 3.81 (dp, *J* = 7.5, 4.1 Hz, 1H, H_{1b}), 3.76-3.70 (m, 1H, $H_{5b'}$), 3.65 – 3.58 (m, 3H, H_{2a} + H_{1a} + H_{5a}), 3.56-3.50 (m, 1H, $H_{5a'}$), 3.02 – 2.91 (m, 2H, H_{3b} + $H_{4b'}$), 2.90 – 2.79 (m, 3H, H_{3a} + H_{4a} + H_{4b}), 2.61 – 2.54 (m, 2H, $H_{3b'}$ + $H_{4a'}$), 2.43 (dd, *J* = 12.3, 10.3 Hz, 1H, $H_{3a'}$), 1.20 (d, *J* = 6.4 Hz, 3H, H_{6b}), 1.14 (d, *J* = 6.3 Hz, 3H, H_{6a}). ^{13}C NMR (126 MHz, $CDCl_3$) δ 77.54 (C_{1a}), 73.41 (C_{2a}), 71.27 (C_{1b}), 67.69 (C_{2b}), 64.43 ($C5_a$+ $C5_b$), 52.47 ($C3_a$), 51.90 ($C3_b$), 47.35 ($C4_a$+ $C4_b$), 19.36 ($C6_a$), 18.52 ($C6_b$). HRMS (*m/z*): calculated for $C_6H_{14}NO_2^+$ $[M+H]^+$: 132.1019, found: 132.1019

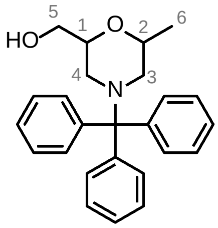

Chemical Formula: $C_{25}H_{27}NO_2$
Molecular Weight: 373.50 g·mol^{-1}

Synthesis of intermediate i. To a solution of (6-methylmorpholin-2-yl)methanol **h** (1.7695 g, 1 equiv, 13.49 mmol) in 30 mL of anhydrous DCM at 0°C was added triethylamine (2.1 mL, 1.1 equiv, 14.84 mmol). In another flask, triphenylmethyl chloride (4.1817 g, 1.1 equiv, 14.84 mmol) was dissolved in 20 mL of anhydrous DCM and this solution was added slowly to the first flask. The mixture was stirred overnight at room temperature. After completion of the reaction, the solvent was removed under vacuum and the crude was dissolved in EtOAc (100 mL), washed with water (50 mL x 3), brine (50 mL x 1) and dried over $MgSO_4$. The crude was purified by column chromatography (SiO_2 deactivated, 350 mL, 18 cm, Cyclohexane/EtOAc 9/1 to 7/3). Fractions containing triphenylmethyl chloride impurities were purified again using the same method yielding the product (3.2056 g, 63%, 8.10 mmol) as white powder. Only one diastereomer described. ^{1}H NMR (500 MHz, $CDCl_3$) δ 7.63 – 7.11 (m, 15H, H_{Ar}), 4.02 (m, 2H, $H_1 + H_2$), 3.54 (ddd, *J* = 11.2, 7.5, 3.5 Hz, 1H, H_5), 3.42 (ddd, *J* = 11.3, 6.8, 4.7 Hz, 1H, $H_{5'}$), 3.01 – 2.91 (m, 2H, $H_3 + H_4$), 1.87 (m, 1H, OH), 1.36 (t, *J* = 10.8 Hz, 1H, $H_{4'}$), 1.25 (dd, *J* = 11.5, 9.9 Hz, 1H, $H_{3'}$), 1.06 (d, *J* = 6.3 Hz, 3H, H_6). ^{13}C NMR (126 MHz, $CDCl_3$) δ 128.99, 128.21, 127.97, 127.44, 126.50, 76.91 (C_1), 72.71 (C_2), 64.69 (C_5), 54.81 (C_3), 49.48 (C_4), 19.50 (C_6). HRMS (*m/z*): calculated for $C_{25}H_{27}NO_2Na^+$ $[M+Na]^+$: 396.1934, found: 396.1933

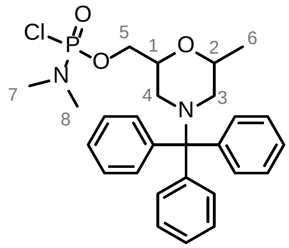

Chemical Formula: $C_{27}H_{32}ClN_2O_3P$
Molecular Weight: 498.99 g·mol^{-1}

Synthesis of 1. (6-methyl-4-tritylmorpholin-2-yl)methanol **i** (304.1 mg, 1 equiv, 8.117x10^{-4}) and LiBr (282.0 mg, 4 equiv, 3.25x10^{-3} mol) were added to a dry flask under argon. 10 mL of dry DCM was added and the flask was cooled at 0°C. DBU (0.18 mL, 1.5 equiv, 1.22x10^{-3} mol). After 5 minutes, $POCl_2NMe_2$ (0.13 mL, 1.3 equiv, 1.05x10^{-3} mol) was added dropwise. After 15 min and the completion of the reaction, the mixture was filtered through a cotton and the solvent was removed. Two columns were done to purify properly the product (SiO_2 deactivated 60 mL, 10 cm, DCM as eluent and SiO_2 deactivated 60 mL, 10 cm, DCM as eluent) yielding the product (150.0 mg, 37 %, 3.01x10^{-4} mol) as a white powder. The two most abundant diastereoisomers are described (a>b). ^{1}H NMR (400 MHz, CD_3CN) δ 7.47 (m, 10H), 7.27 (m, 12H), 7.18 (m, 6H), 4.14 (m, 1.7H, $H_{1a/b}$), 4.04 (m, 5H, H2a/b, $H_{5a/b}$), 3.03 (m, 1.56H, $H_{4a/b}$), 2.97 (m, 1.63H, $H_{3a/b}$), 2.60 (s, 3H, $H_{7/8a}$), 2.59

(s, 1.52H, $H_{7/8b}$), 2.56 (s, 3H, $H_{7/8a}$), 2.55 (s, 1.52H, $H_{7/8b}$), 1.28 (m, 2.24H, $H_{4a/b}$), 1.13 (m, 1.83H, $H_{3a/b}$), 0.98 (d, J = 6.3 Hz, 3.28H, $H_{6a/b}$). C1 is not well defined. ^{13}C NMR (101 MHz, CD_3CN) δ 148.43, 130.34, 128.58, 127.25, 75.01 (C_1), 74.79 (C_1), 73.02 (C_{2b}), 72.98 (C_{2a}), 69.21 (C_{5a}), 69.15 (C_{5b}), 55.34 (C_{3a}), 55.25 (C_{3b}), 50.37 (C_{4a}), 50.13(C_{4b}), 36.86($C_{7/8\ a/b}$), 19.28 ($C_{6a/b}$). HRMS (*m/z*): calculated for $C_{27}H_{32}N_2O_3PClNa^+$ $[M+Na]^+$: 521.1731, found: 521.1738

A.4. Resin modification. The detailed procedure for resin modification is shown in Scheme 3 of the main document. Corresponding NMR spectra are shown in Figures S38-S39.

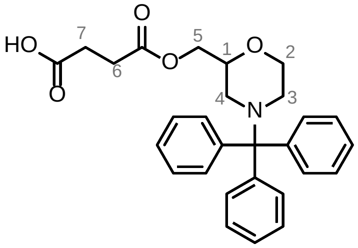

Chemical Formula: $C_{28}H_{29}NO_5$
Molecular Weight: 459.54 $g{\cdot}mol^{-1}$

Synthesis of intermediate j. To a solution of (4-tritylmorpholin-2-yl)methanol **e** (3.3320 g, 1 equiv, 9.26 mmol) in 10 mL of dry pyridine at 0 °C, DMAP (0.113 g, 0.1 equiv, 0.926 mmol) and succinic anhydride (1.85 g, 2 equiv, 18.5 mmol) were added. The color of the reaction turned to white upon the addition of succinic anhydride. After two hours, the mixture is colorless and the TLC indicated the consumption of the starting material. The solvent was removed under vacuum and the crude compound was purified by column chromatography (SiO_2, 300 mL, 17 cm, EtOAc / Cyclohexane 3/7). The NMR analysis revealed some residual pyridine in the product. The product was dissolved in 50 mL of EtOAc and was washed with a 10% $CuSO_4$ solution (3 x 10 mL), water (5 x 10 mL), brine (1 x 10 mL) and dried over $MgSO_4$ yielding the product (3.16 g, 74%, 6.87 mmol) as a white powder. To avoid broad signals in ^{1}H NMR analysis, the temperature of the spectrometer was cooled at 253 K. ^{1}H NMR (400 MHz, $CDCl_3$) δ 7.50-7.43 (m , 6H, H_{Ar}), 7.31-7.23 (m, 6H, H_{Ar}), 7.20-7.13 (m, 3H, H_{Ar}), 4.08 – 3.84 (m, 5H, $H_1 + H_5 + H_2 + H_{5'} + H_{2'}$), 2.92 (m, 2H, $H_3 + H_4$), 2.62 (m, 4H, 2 x H_6 + 2 x H_7), 1.64 (t, J = 11.5 Hz, 1H, H_3), 1.38 (t, J = 10.8 Hz, 1H, H_4). ^{13}C NMR (101 MHz, $CDCl_3$) δ 172.31, 129.70, 127.99, 126.59, 74.37 (C_1), 67.50 (C_2), 65.86 (C_5), 50.22 (C_4), 48.29 (C_3), 29.09 (C_6 + C_7). HRMS (*m/z*): calculated for $C_{28}H_{29}NO_5Na^+$ $C_{28}H_{29}O_5N$ $[M+Na]^+$: 482.1938 , found: 482.1933

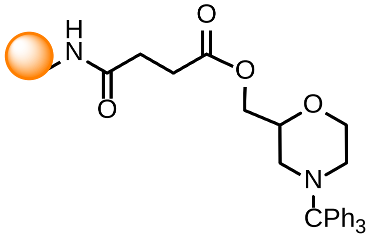

Resin functionalization. 15 mg of aminomethylated polystyrene resin (1 equiv, 1.25mmol/g, 1.875 x 10^{-5} mol) was introduced into a SPE tube and swollen with DCM (2 x 20 min). DCC (38 mg, 10 equiv, 1.875 x 10^{-4} mol), DMAP (6 mg, 2 equiv, 3.75 x 10^{-5} mol)

and **j** (26 mg, 3 equiv, 5.63 x 10^{-5} mol) were then introduced with 2 mL of DCM and the resin was swell overnight at RT.

A.5. Solid phase synthesis of PMOs. The general strategy for the solid-phase synthesis of PMOs is shown in Scheme 4 of the main document. Directly after its functionalization, the modified polystyrene resin (1 equiv, 1.25 mmol/g, 1.875 x 10^{-5} mol) was kept into a SPE tube and swollen overnight in DCM at RT (see resin functionalization in section A.4). The next day, the resin was washed several times with DCM and NMP. For the trityl deprotection step *(i)*, 2 mL of TFA in DCM 20% was added to the resin. A yellow-orange color was formed and the tube was shaken. After 10 min, the acidic solution was removed and 2 mL of the deprotection solution was added again. This operation was repeated 3 times, once 10 min and twice 5 min. At the end of the deprotection step, the resin was washed one time with DCM and swollen (3 x 5 min) with a basic solution (DIPEA in NMP 20%). For the coupling step *(ii)*, the resin was washed with DCM and swollen for 20 min with NMP. The monomer (**0** or **1**) (4 equiv, 7.5 x 10^{-5} mol) was dissolved in 1 mL of NMP with NEM (23.7 µL, 10 equiv, 1.875 x 10^{-4} mol) and ETT (0.8 mL, 0.25 M in MeCN, 10 equiv). This solution was stirred with the resin (3 x 40 min). For the capping step *(iii)*, the resin was washed with DCM and capped with 1 ml of Ac_2O in NMP (20/80) and 1 ml of NMI in NMP (40/80) (5 x 10 min). The resin was finally washed with DCM. Steps *(i)*, *(ii)* and *(iii)* were repeated a certain number of times until a desired oligomer length was reached. For some oligomers only steps *(i)* and *(ii)* were used (see main text for details). For cleavage *(iv)*, after the last capping step, the resin was swollen in water (2 x 20 min) and introduced into a flask with 2 or 3 mL of NH_4OH and stirred overnight at 60-65 °C. The cleavage solution was then purified on a DNA reverse phase cartridge. The solution was mixed with 1 mL of NaCl in water (100 mg/mL). The column was conditioned with 1 mL of MeCN and 1 mL of buffer solution (2.0 M of TEEA). The cleavage solution was added to the column and washed (10 mL with 20% MeCN in 100 mg/mL sodium chloride). Finally, the trityl group of the oligomers was removed with TFA (10% in water, 6 mL). The cartridge was washed with MiliQ water (10 mL) and the pure oligomer was eluted (2 mL of 50% MeCN/H_2O containing 0.5% ammonium hydroxide).

B. Measurements and analyses.

B.1. Nuclear Magnetic Resonance. NMR data were recorded on a Bruker Avance III 400 spectrometer or Bruker Avance Neo 500 MHz. Coupling constants (*J*) are listed in hertz (Hz). Chemical shifts (δ) are reported in parts per million (ppm) against solvent residual signal (^{1}H NMR, $CDCl_3$: δ = 7.26 ppm; ^{13}C NMR, $CDCl_3$: δ = 77.16 ppm; ^{1}H NMR, CD_3CN: δ = 2.08 ppm; ^{13}C NMR, CD_3CN: δ = 118.26 and 1.79 ppm. The following notation is used for the ^{1}H NMR spectral splitting patterns: singlet (s), doublet (d), triplet (t), quartet (q), quintet (quint), multiplet (m), broad (br), doublet of doublets (dd), doublet of triplets (dt), doublet of doublets of doublets (ddd), triplet of doublets (td). Two hydrogens on the carbon can be name as H and H')

B.2. Mass Spectrometry. All MS and MS/MS experiments were performed with a ZenoTOF mass spectrometer (Sciex, Concord, Ontario, Canada). Sample solutions (50% ACN/H_2O containing 0.5% ammonium hydroxide) were diluted (dilution factor: 1/10 to 1/100) in supplemented with ammonium acetate (3 mM) and introduced with a syringe pump at a 10 μL min^{-1} flow rate in the electrospray ionization (ESI) source operated in the positive mode (capillary voltage: +5.5 kV) under a nebulizing gas (air, 20 psi) heated at 35°C. The cone voltage was +75 V. In this hybrid instrument, ions were measured using an orthogonal acceleration time-of-flight (oa-TOF) mass analyzer. In the MS/MS mode, a quadrupole was used for selection of precursor ions to be further submitted to collision-induced dissociation (CID) in a collision cell filled with N_2. Accurate mass measurements were performed using internal calibration. All spectra were recorded for 0.1 min, corresponding to 22 scans. Instrument control, data acquisition and data processing of all experiments were achieved using Sciex OS software (3.4.0) provided by Sciex.

C. Additional data and figures.

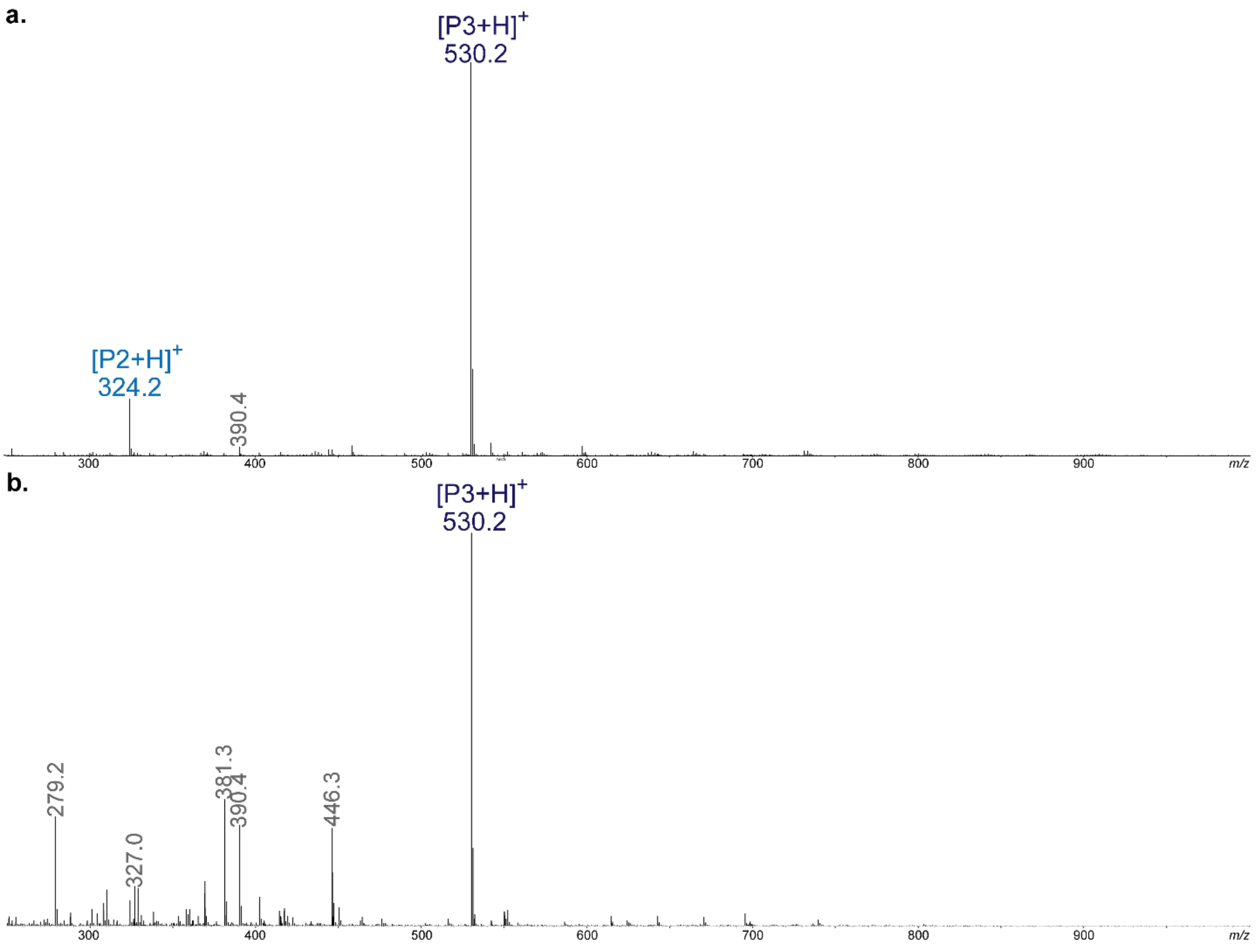

Figure S1. ESI-HRMS of oligomer **P3** (α00, 529.2 Da), detected as a protonated molecule at *m/z* 530.2 prepared (**a**) without or (**b**) with capping steps. In grey: chemical background of the ESI source.

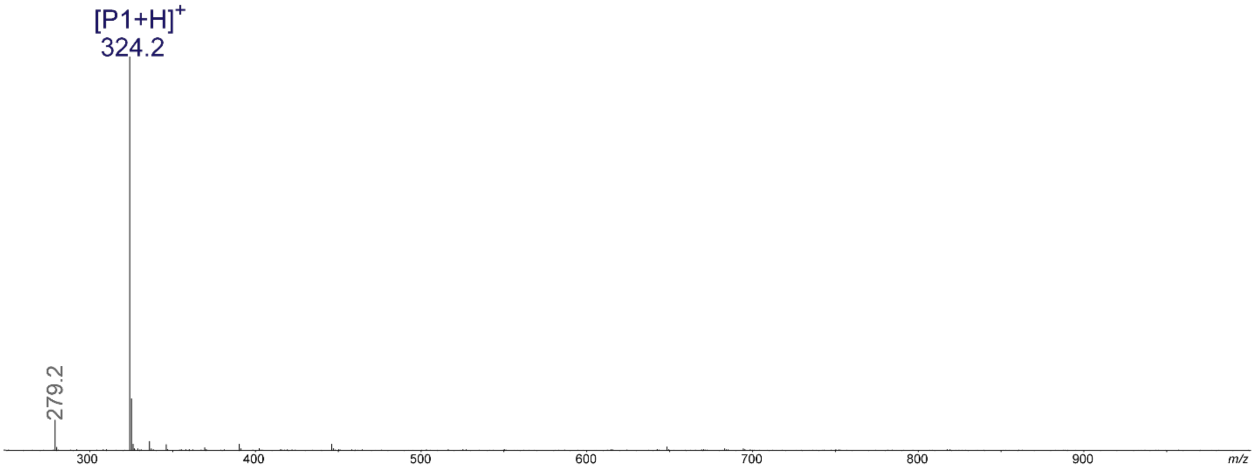

Figure S2. ESI-HRMS of oligomer **P1** (α0, 323.2 Da), detected as a protonated molecule at *m/z* 324.2. In grey: chemical background of the ESI source.

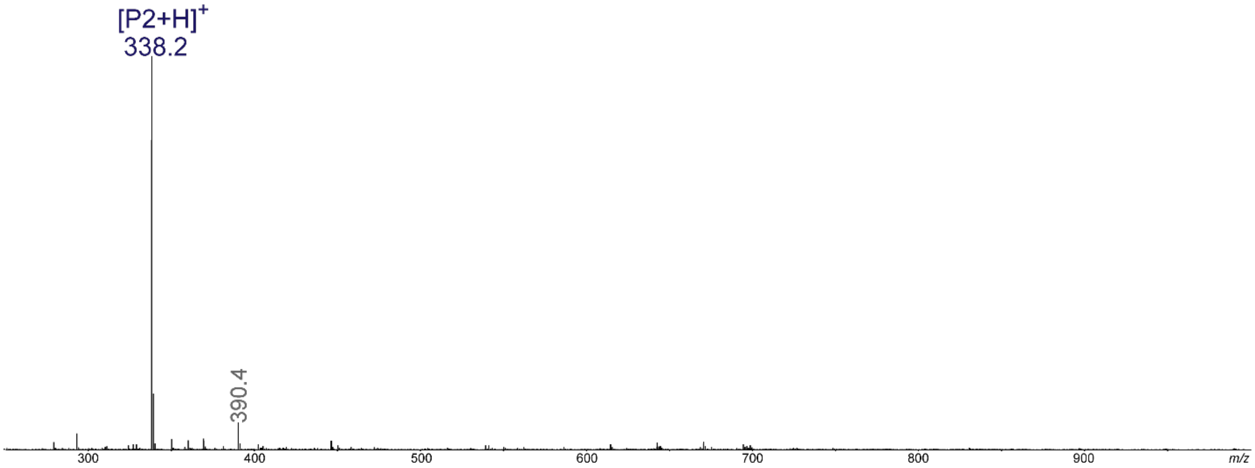

Figure S3. ESI-HRMS of oligomer **P2** (α1, 337.2 Da), detected as a protonated molecule at *m/z* 338.2. In grey: chemical background of the ESI source.

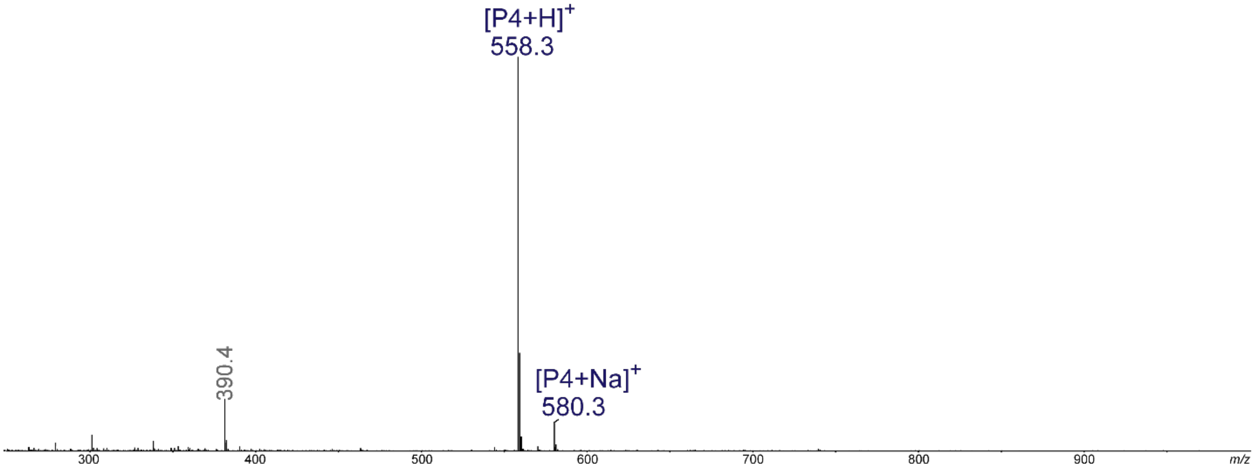

Figure S4. ESI-HRMS of oligomer **P4** (α11, 557.3 Da), detected as a protonated molecule at *m/z* 557.3 and as a sodium adduct at *m/z* 580.3. In grey: chemical background of the ESI source.

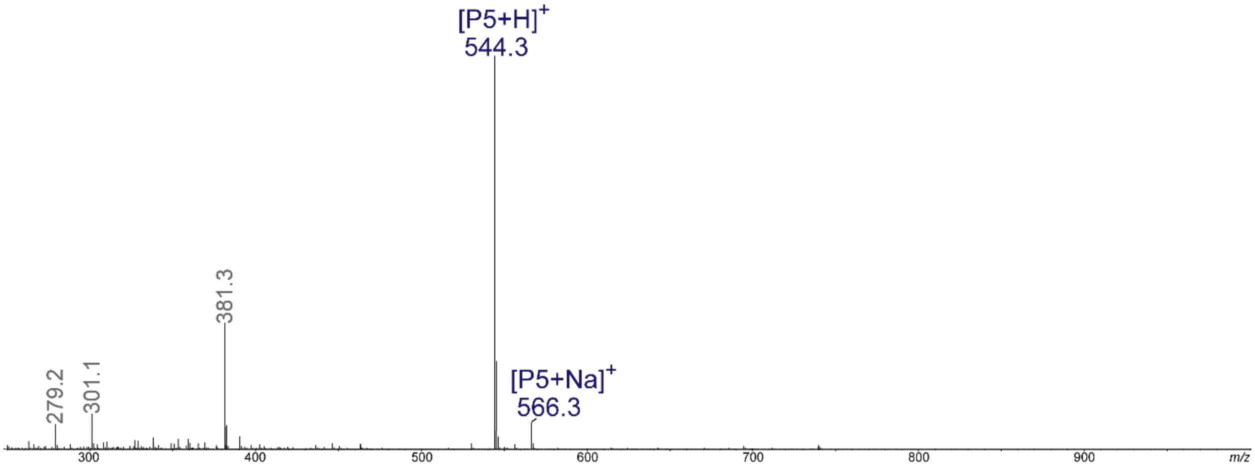

Figure S5. ESI-HRMS of oligomer **P5** (α01, 543.3 Da), detected as a protonated molecule at *m/z* 544.3 and as a sodium adduct at *m/z* 566.3. In grey: chemical background of the ESI source.

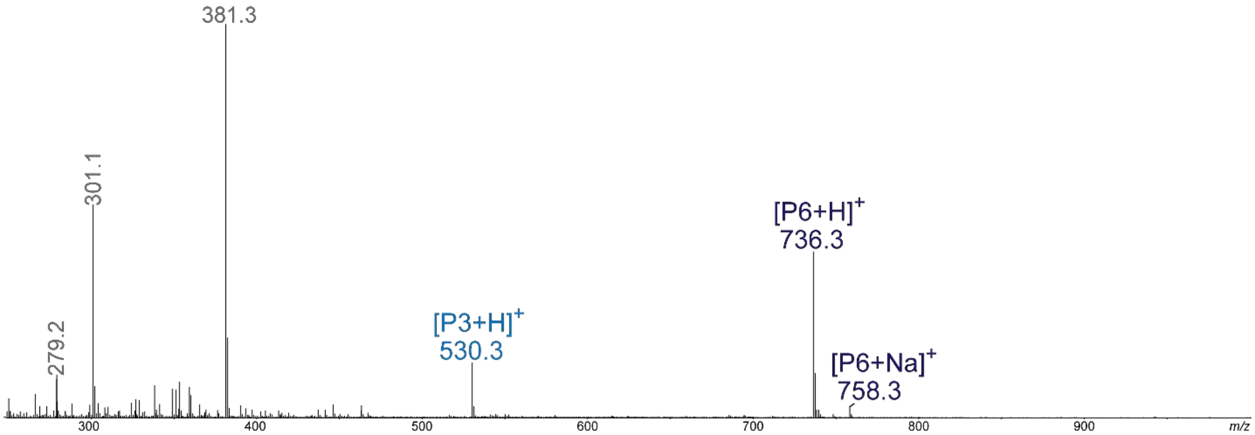

Figure S6. ESI-HRMS of oligomer **P6** (α000, 735.3 Da), detected as a protonated molecule at *m/z* 736.3 and as a sodium adduct at *m/z* 758.3. MS/MS data recorded for the *m/z* 530.3 ion permit to reveal the α00 sequence of **P3**. In grey: chemical background of the ESI source.

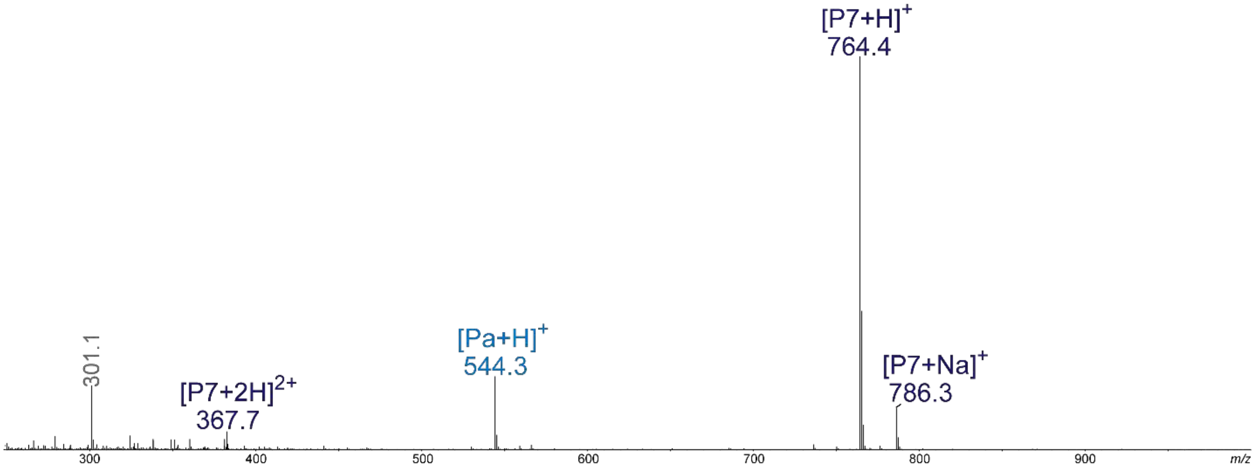

Figure S7. ESI-HRMS of oligomer **P7** (α110, 763.4 Da), detected as a singly (*m/z* 764.4) and doubly (*m/z* 367.7) protonated molecule and as a singly charged sodium adduct (*m/z* 786.3). MS/MS data recorded for the *m/z* 544.3 ion permit to reveal the α10 sequence of **Pa**. In grey: chemical background of the ESI source.

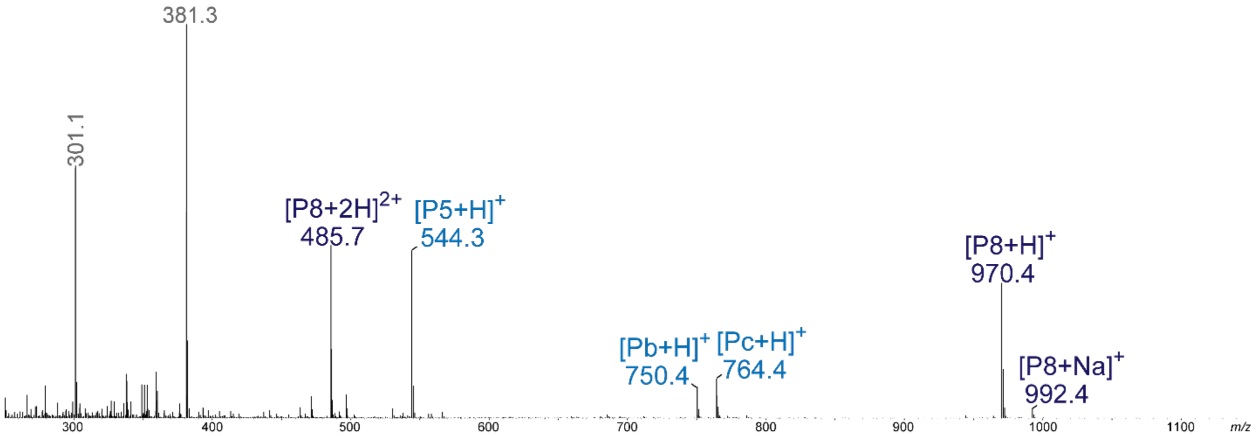

Figure S8. ESI-HRMS of oligomer **P8** (α0101, 969.4 Da), detected as a singly (*m/z* 970.4) and doubly (*m/z* 485.7) protonated molecule and as a singly charged sodium adduct (*m/z* 992.4). MS/MS data permit to reveal the sequence of protonated oligomers detected at *m/z* 544.3 (**P5**: α01), *m/z* 750.4 (**Pb**: α001) and *m/z* 764.4 (**Pc**: α011). In grey: chemical background of the ESI source.

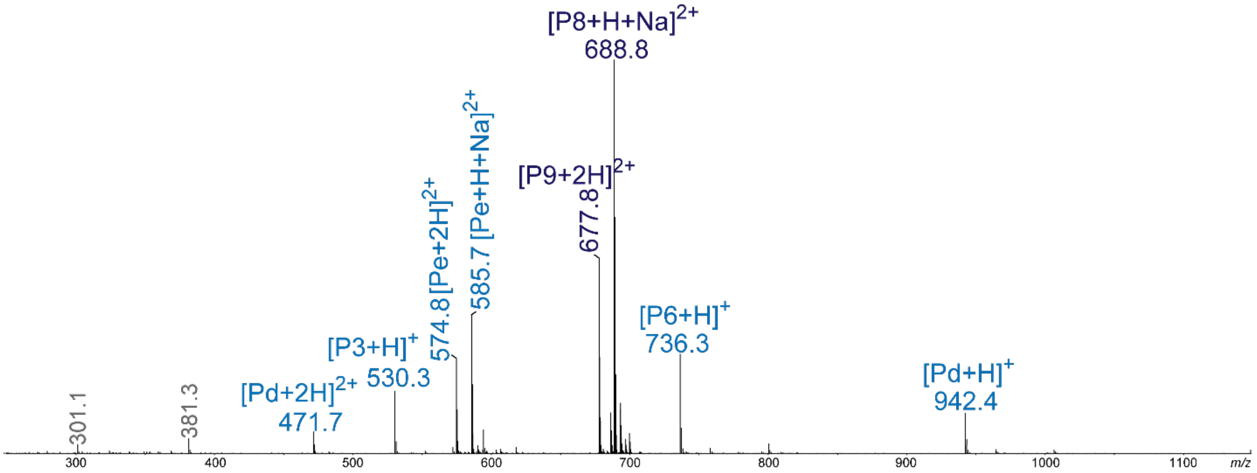

Figure S9. ESI-HRMS of oligomer **P9** (α000000, 1353.6 Da), detected as a doubly protonated molecule at *m/z* 677.8 and as the doubly charged mixed adduct $[P8+H+Na]^{2+}$ at *m/z* 688.8. MS/MS data permit to validate the sequence of other oligomers present in this sample: **P3** (α00), **P6** (α000), **Pd** (α0000) and **Pe** (α00000). In grey: chemical background of the ESI source.

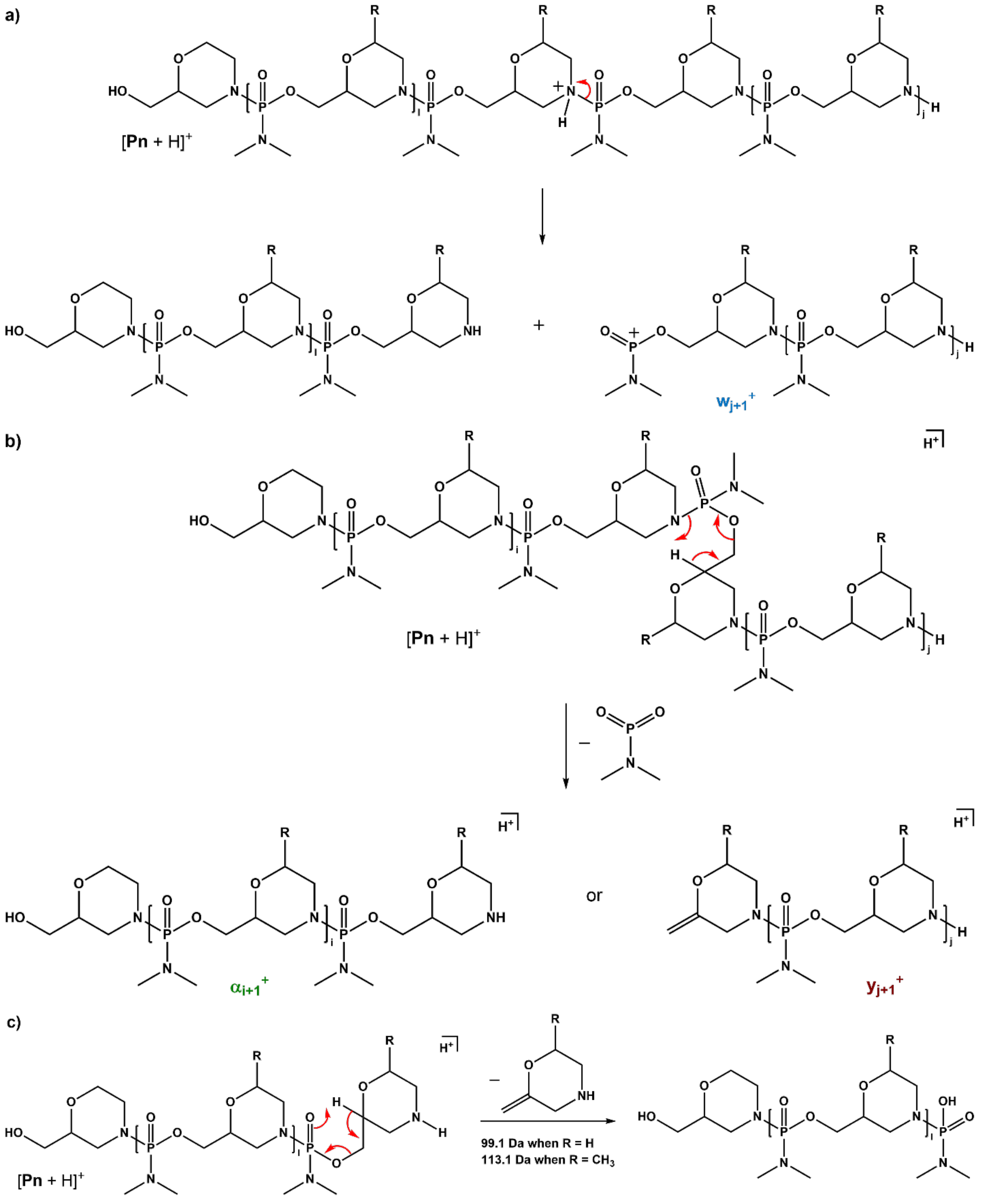

Scheme S1. Proposed mechanisms for the formation of a) w^+ fragments, b) α^+ and y^+ fragments, and c) product ions generated after elimination of the last coding morpholine ring.

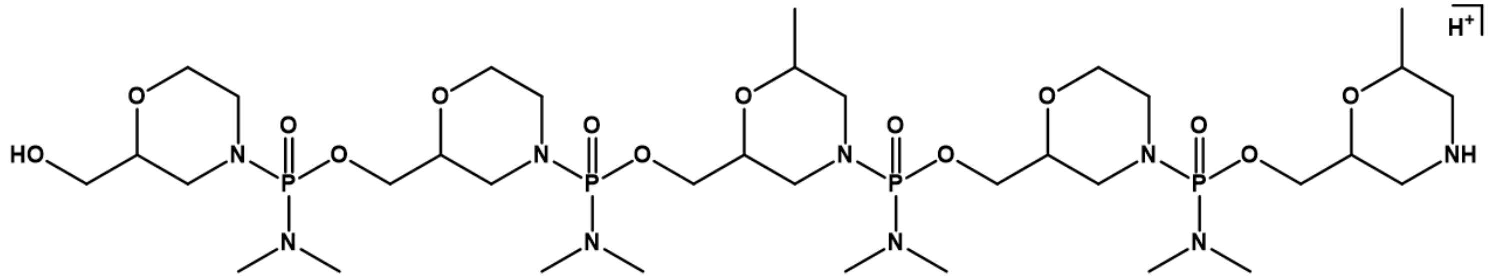

[**P8** + H]$^+$		★		★★		★★★		★★★★
$C_{35}H_{76}N_9O_{14}P_4^+$	*– 45 Da*	$C_{33}H_{69}N_8O_{14}P_4^+$	*– 45 Da*	$C_{31}H_{62}N_7O_{14}P_4^+$	*– 45 Da*	$C_{29}H_{55}N_6O_{14}P_4^+$	*– 45 Da*	$C_{27}H_{48}N_5O_{14}P_4^+$
m/z_{calc} 970.4457	→	m/z_{calc} 925.3878	→	m/z_{calc} 880.3300	→	m/z_{calc} 835.2721	→	m/z_{calc} 790.2143
I.S.		m/z_{exp} 925.3869		m/z_{exp} 880.3289		m/z_{exp} 835.2712		m/z_{exp} 790.2155
↓ *– 113 Da*		↓ *– 113 Da*		↓ *– 113 Da*		↓ *– 113 Da*		↓ *– 113 Da*
●		●★		●★★		●★★★		●★★★★
$C_{29}H_{65}N_8O_{13}P_4^+$	*– 45 Da*	$C_{27}H_{58}N_7O_{13}P_4^+$	*– 45 Da*	$C_{25}H_{51}N_6O_{13}P_4^+$	*– 45 Da*	$C_{23}H_{44}N_5O_{13}P_4^+$	*– 45 Da*	$C_{21}H_{37}N_4O_{13}P_4^+$
m/z_{calc} 857.3616	→	m/z_{calc} 812.3038	→	m/z_{calc} 767.2459	→	m/z_{calc} 722.1881	→	m/z_{calc} 722.1881
m/z_{exp} 857.3607		m/z_{exp} 812.3030		m/z_{exp} 767.2451		m/z_{exp} 722.1880		m/z_{exp} 722.1880

Table S1. Accurate mass measurements of fragments obtained after protonated **P8** (α0101, top structure) has released neutrals of mass 45 Da (C_2H_7N), 113 Da ($C_6H_{11}NO$), and combination thereof, in the CID spectrum shown in Figure 2. *I.S.*: internal standard.

α_0^+<br>$C_5H_{12}NO_2^+$<br>m/z_{calc} 118.0863<br>m/z_{exp} 118.0862						
α_1^+<br>$C_{12}H_{27}N_3O_5P^+$<br>m/z_{calc} 324.1683<br>m/z_{exp} 324.1683	*– 45 Da* →	★<br>$C_{10}H_{20}N_2O_5P^+$<br>m/z_{calc} 279.1104<br>m/z_{exp} 279.1105				
↓ *– 99 Da*		↓ *– 99 Da*				
■<br>$C_7H_{18}N_2O_4P^+$<br>m/z_{calc} 225.0999<br>m/z_{exp} 225.1001	*– 45 Da* →	■★<br>$C_5H_{11}NO_4P^+$<br>m/z_{calc} 180.0420<br>m/z_{exp} 180.0416				
α_2^+<br>$C_{20}H_{44}N_5O_8P_2^+$<br>m/z_{calc} 544.2660<br>m/z_{exp} 544.2658	*– 45 Da* →	★<br>$C_{18}H_{37}N_4O_8P_2^+$<br>m/z_{calc} 499.2081<br>m/z_{exp} 499.2078	*– 45 Da* →	★★<br>$C_{16}H_{30}N_3O_8P_2^+$<br>m/z_{calc} 454.1503<br>m/z_{exp} 454.1501		
↓ *– 113 Da*		↓ *– 113 Da*		↓ *– 113 Da*		
●<br>$C_{14}H_{33}N_4O_7P_2^+$<br>m/z_{calc} 431.1819<br>m/z_{exp} 431.1814	*– 45 Da* →	●★<br>$C_{12}H_{26}N_3O_7P_2^+$<br>m/z_{calc} 386.1241<br>m/z_{exp} 386.1240	*– 45 Da* →	●★★<br>$C_{10}H_{19}N_2O_7P_2^+$<br>m/z_{calc} 341.0662<br>m/z_{exp} 341.0662		
α_3^+<br>$C_{27}H_{59}N_7O_{11}P_3^+$<br>$m/z_{calc}$ 750.3480<br>m/z_{exp} 750.3477	*– 45 Da* →	★<br>$C_{25}H_{52}N_6O_{11}P_3^+$<br>$m/z_{calc}$ 705.2901<br>m/z_{exp} 705.2894	*– 45 Da* →	★★<br>$C_{23}H_{45}N_5O_{11}P_3^+$<br>$m/z_{calc}$ 660.2323<br>m/z_{exp} 660.2318	*– 45 Da* →	★★★<br>$C_{21}H_{38}N_4O_{11}P_3^+$<br>$m/z_{calc}$ 615.1745<br>m/z_{exp} 615.1732
↓ *– 99 Da*		↓ *– 99 Da*		↓ *– 99 Da*		↓ *– 99 Da*
■<br>$C_{22}H_{50}N_6O_{10}P_3^+$<br>$m/z_{calc}$ 651.2796<br>m/z_{exp} 651.2788	*– 45 Da* →	■★<br>$C_{20}H_{43}N_5O_{10}P_3^+$<br>$m/z_{calc}$ 606.2217<br>m/z_{exp} 606.2214	*– 45 Da* →	■★★<br>$C_{18}H_{36}N_4O_{10}P_3^+$<br>$m/z_{calc}$ 561.1639<br>m/z_{exp} 561.1639	*– 45 Da* →	■★★★<br>$C_{16}H_{29}N_3O_{10}P_3^+$<br>$m/z_{calc}$ 516.1060<br>m/z_{exp} 516.1063

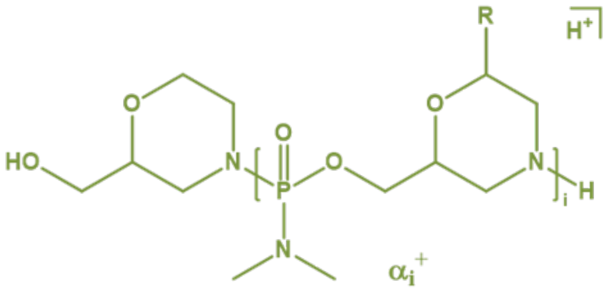

Table S2. Accurate mass measurements of α_i^+ ions (structure in inset) and of their fragments formed after loss of neutrals of mass 45 Da (C_2H_7N), 99 Da (C_5H_9NO), 113 Da ($C_6H_{11}NO$), and combination thereof, in the CID spectrum of protonated **P8** (α0101) shown in Figure 2.

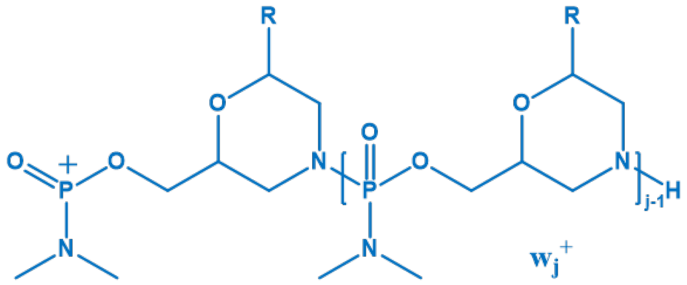

w_1^+<br>$C_8H_{18}N_2O_3P^+$<br>m/z_{calc} 221.1050<br>m/z_{exp} 221.1050	*– 45 Da* →	★<br>$C_6H_{11}N_2O_3P^+$<br>m/z_{calc} 176.0471<br>m/z_{exp} 176.0471						
w_2^+<br>$C_{15}H_{33}N_4O_6P_2^+$<br>m/z_{calc} 427.1870<br>m/z_{exp} 427.1869	*– 45 Da* →	★<br>$C_{13}H_{26}N_3O_6P_2^+$<br>m/z_{calc} 382.1291<br>m/z_{exp} 382.1292	*– 45 Da* →	★★<br>$C_{11}H_{19}N_2O_6P_2^+$<br>m/z_{calc} 337.0713<br>m/z_{exp} 337.0713				
↓ *– 113 Da*		↓ *– 113 Da*		↓ *– 113 Da*				
●<br>$C_9H_{22}N_3O_5P_2^+$<br>m/z_{calc} 314.1029<br>m/z_{exp} 314.1030	*– 45 Da* →	●★<br>$C_7H_{15}N_2O_5P_2^+$<br>m/z_{calc} 269.0451<br>m/z_{exp} 269.0452	*– 45 Da* →	●★★<br>$C_5H_8NO_5P_2^+$<br>m/z_{calc} 223.9872<br>m/z_{exp} 223.9857				
w_3^+<br>$C_{23}H_{50}N_6O_9P_3^+$<br>m/z_{calc} 647.2847<br>m/z_{exp} 647.2846	*– 45 Da* →	★<br>$C_{21}H_{43}N_5O_9P_3^+$<br>m/z_{calc} 602.2268<br>m/z_{exp} 602.2266	*– 45 Da* →	★★<br>$C_{19}H_{36}N_4O_9P_3^+$<br>m/z_{calc} 557.1650<br>m/z_{exp} 557.1686	*– 45 Da* →	★★★<br>$C_{17}H_{29}N_3O_9P_3^+$<br>m/z_{calc} 512.1111<br>m/z_{exp} 512.1103		
↓ *– 113 Da*		↓ *– 113 Da*		↓ *– 113 Da*		↓ *– 113 Da*		
●<br>$C_{17}H_{39}N_5O_8P_3^+$<br>m/z_{calc} 534.2006<br>m/z_{exp} 534.2001	*– 45 Da* →	●★<br>$C_{15}H_{32}N_4O_8P_3^+$<br>m/z_{calc} 489.1428<br>m/z_{exp} 489.1426	*– 45 Da* →	●★★<br>$C_{13}H_{25}N_3O_8P_3^+$<br>m/z_{calc} 444.0849<br>m/z_{exp} 444.0851	*– 45 Da* →	●★★★<br>$C_{11}H_{18}N_2O_8P_3^+$<br>m/z_{calc} 399.0271<br>m/z_{exp} 399.0269		
w_4^+<br>$C_{30}H_{65}N_8O_{12}P_4^+$<br>$m/z_{calc}$ 853.3667<br>m/z_{exp} 853.3656	*– 45 Da* →	★<br>$C_{28}H_{58}N_7O_{12}P_4^+$<br>$m/z_{calc}$ 808.3089<br>m/z_{exp} 808.3075	*– 45 Da* →	★★<br>$C_{26}H_{51}N_6O_{12}P_4^+$<br>$m/z_{calc}$ 763.2510<br>m/z_{exp} 763.2497	*– 45 Da* →	★★★<br>$C_{24}H_{44}N_5O_{12}P_4^+$<br>$m/z_{calc}$ 718.1932<br>m/z_{exp} 718.1937	*– 45 Da* →	★★★★<br>$C_{22}H_{37}N_4O_{12}P_4^+$<br>$m/z_{calc}$ 673.1353<br>*n.d.*
↓ *– 113 Da*		↓ *– 113 Da*		↓ *– 113 Da*		↓ *– 113 Da*		↓ *– 113 Da*
●<br>$C_{24}H_{54}N_7O_{11}P_4^+$<br>$m/z_{calc}$ 740.2826<br>m/z_{exp} 740.2821	*– 45 Da* →	●★<br>$C_{22}H_{47}N_6O_{11}P_4^+$<br>$m/z_{calc}$ 695.2248<br>m/z_{exp} 695.2244	*– 45 Da* →	●★★<br>$C_{20}H_{40}N_5O_{11}P_4^+$<br>$m/z_{calc}$ 650.1669<br>m/z_{exp} 650.1684	*– 45 Da* →	●★★★<br>$C_{18}H_{33}N_4O_{11}P_4^+$<br>$m/z_{calc}$ 605.1091<br>m/z_{exp} 605.1102	*– 45 Da* →	●★★★★<br>$C_{16}H_{26}N_3O_{11}P_4^+$<br>$m/z_{calc}$ 560.0512<br>*n.d.*

Table S3. Accurate mass measurements of w_j^+ ions (structure in inset) and of their fragments formed after loss of neutrals of mass 45 Da (C_2H_7N), 113 Da ($C_6H_{11}NO$), and combination thereof, in the CID spectrum of protonated **P8** (α0101) shown in Figure 2. *n.d.*: not detected.

**y_1^+**<br>$C_6H_{12}NO^+$<br>m/z_{calc} 114.0913<br>m/z_{exp} 114.0914						
**y_2^+**<br>$C_{13}H_{27}N_3O_4P^+$<br>m/z_{calc} 320.1734<br>m/z_{exp} 320.1735	*– 45 Da*<br>→	★<br>$C_{11}H_{20}N_2O_4P^+$<br>m/z_{calc} 275.1155<br>m/z_{exp} 275.1156				
↓ *– 113 Da*		↓ *– 113 Da*				
●<br>$C_7H_{16}N_2O_3P^+$<br>m/z_{calc} 207.0893<br>m/z_{exp} 207.0894	*– 45 Da*<br>→	●★<br>$C_5H_9NO_3P^+$<br>m/z_{calc} 162.0315<br>m/z_{exp} 162.0315				
**y_3^+**<br>$C_{21}H_{44}N_5O_7P_2^+$<br>m/z_{calc} 540.2711<br>m/z_{exp} 540.2707	*– 45 Da*<br>→	★<br>$C_{19}H_{37}N_4O_7P_2^+$<br>m/z_{calc} 495.2132<br>m/z_{exp} 495.2131	*– 45 Da*<br>→	★★<br>$C_{17}H_{30}N_3O_7P_2^+$<br>m/z_{calc} 450.1554<br>m/z_{exp} 450.1554		
↓ *– 113 Da*		↓ *– 113 Da*		↓ *– 113 Da*		
●<br>$C_{15}H_{33}N_4O_6P_2^+$<br>m/z_{calc} 427.1870<br>m/z_{exp} 427.1869	*– 45 Da*<br>→	●★<br>$C_{13}H_{26}N_3O_6P_2^+$<br>m/z_{calc} 382.1291<br>m/z_{exp} 382.1292	*– 45 Da*<br>→	●★★<br>$C_{11}H_{19}N_2O_6P_2^+$<br>m/z_{calc} 337.0713<br>m/z_{exp} 337.0713		
**y_4^+**<br>$C_{28}H_{59}N_7O_{10}P_3^+$<br>$m/z_{calc}$ 746.3531<br>m/z_{exp} 746.3524	*– 45 Da*<br>→	★<br>$C_{26}H_{52}N_6O_{10}P_3^+$<br>$m/z_{calc}$ 701.2952<br>m/z_{exp} 701.2948	*– 45 Da*<br>→	★★<br>$C_{24}H_{45}N_5O_{10}P_3^+$<br>$m/z_{calc}$ 656.2374<br>m/z_{exp} 656.2370	*– 45 Da*<br>→	★★★<br>$C_{22}H_{38}N_4O_{10}P_3^+$<br>$m/z_{calc}$ 611.1795<br>m/z_{exp} 611.1790
↓ *– 113 Da*		↓ *– 113 Da*		↓ *– 113 Da*		↓ *– 113 Da*
●<br>$C_{22}H_{48}N_6O_9P_3^+$<br>m/z_{calc} 633.2690<br>m/z_{exp} 633.2683	*– 45 Da*<br>→	●★<br>$C_{20}H_{41}N_5O_9P_3^+$<br>m/z_{calc} 588.2112<br>m/z_{exp} 588.2109	*– 45 Da*<br>→	●★★<br>$C_{18}H_{34}N_4O_9P_3^+$<br>m/z_{calc} 543.1533<br>m/z_{exp} 543.1532	*– 45 Da*<br>→	●★★★<br>$C_{16}H_{27}N_3O_9P_3^+$<br>m/z_{calc} 498.0955<br>m/z_{exp} 498.0950

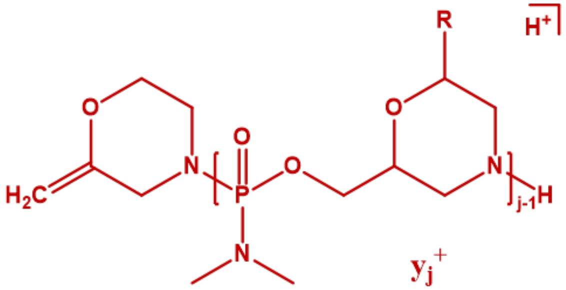

Table S4. Accurate mass measurements of y_j^+ ions (structure in inset) and of their fragments formed after loss of neutrals of mass 45 Da (C_2H_7N), 113 Da ($C_6H_{11}NO$), and combination thereof, in the CID spectrum of protonated **P8** (α0101) shown in Figure 2.

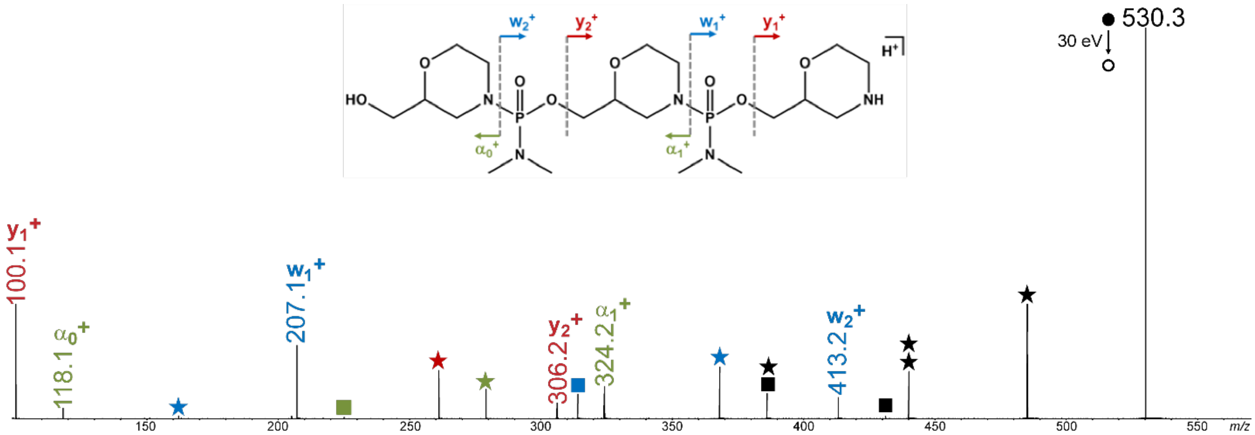

Figure S10. MS/MS of [**P3** + H]$^+$ at *m/z* 530.3 (inset structure). Symbols designate fragments obtained after loss of dimethylamine (45 Da, star) or 2-methylenemorpholine (99 Da, square), and combination thereof, from the precursor ion (black) or fragments α^+ (green), w^+ (blue) and y^+ (red).

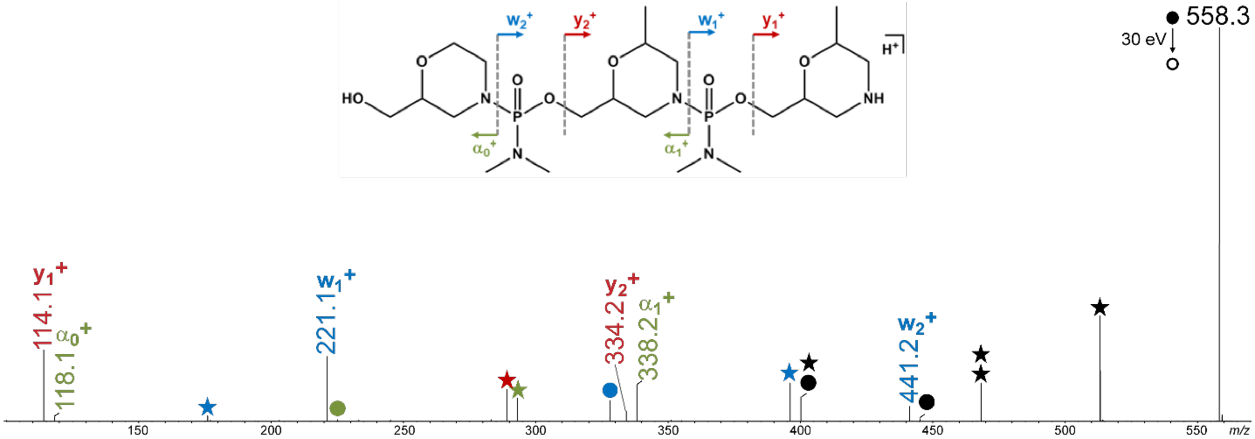

Figure S11. MS/MS of [**P4** + H]$^+$ at *m/z* 558.3 (inset structure). Symbols designate fragments forme after loss of dimethylamine (45 Da, star) or 2-methylene-6-methylmorpholine (113 Da, circle), and combination thereof, from the precursor ion (black) or fragments α^+ (green), w^+ (blue) and y^+ (red).

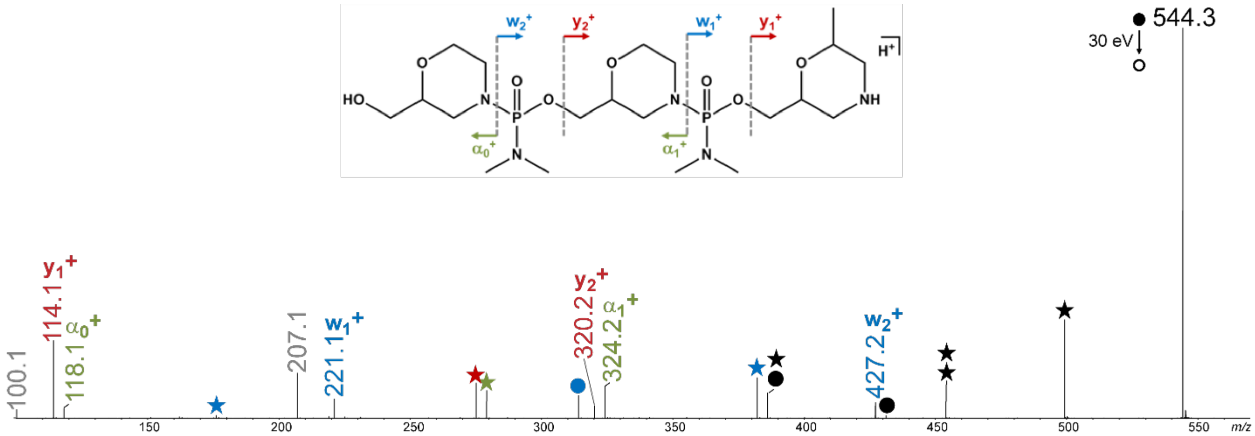

Figure S12. MS/MS of [**P5** + H]$^+$ at *m/z* 544.3 (inset structure). Symbols designate ions formed after loss of dimethylamine (45 Da, star) or 2-methylene-6-methylmorpholine (113 Da, circle), and combination thereof, from the precursor ion (black) or primary fragments α^+ (green), w^+ (blue) and y^+ (red). Other internal fragments are in grey.

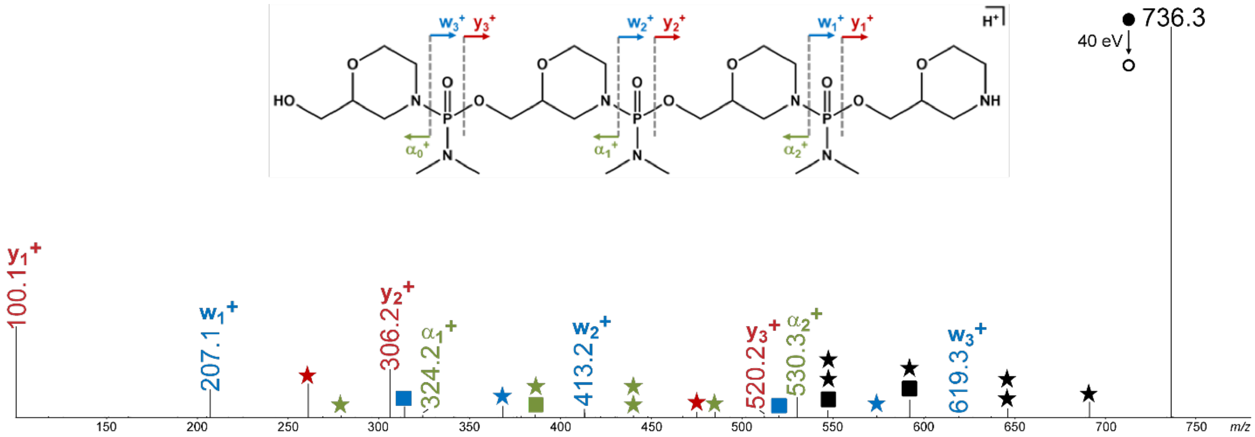

Figure S13. MS/MS of [**P6** + H]$^+$ at *m/z* 736.3 (inset structure). Symbols designate ions formed after loss of dimethylamine (45 Da, star) or 2-methylenemorpholine (99 Da, square) and combination thereof, from the precursor ion (black) or primary fragments α$^+$ (green), w$^+$ (blue) and y$^+$ (red).

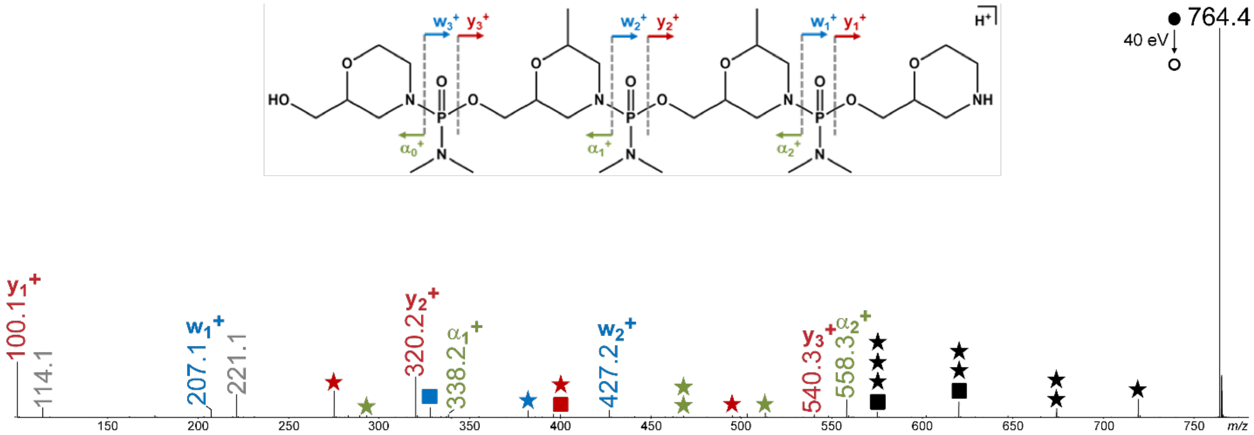

Figure S14. MS/MS of [**P7** + H]$^+$ at *m/z* 764.4 (inset structure). Symbols designate ions formed after loss of dimethylamine (45 Da, star) or 2-methylenemorpholine (99 Da, square) and combination thereof, from the precursor ion (black) or primary fragments α$^+$ (green), w$^+$ (blue) and y$^+$ (red). Other internal fragments are in grey.

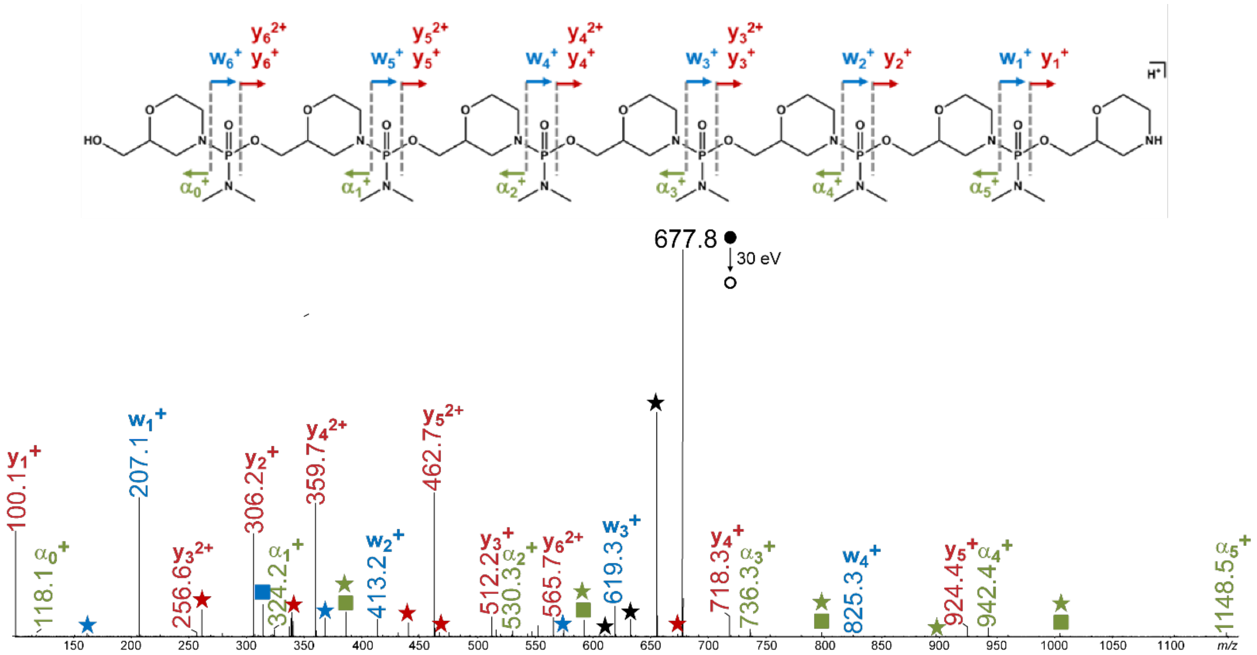

Figure S15. MS/MS of [**P9** + 2H]$^{2+}$ at *m/z* 677.8 (inset structure). Symbols designate ions formed after loss of dimethylamine (45 Da, star) or 2-methylenemorpholine (99 Da, square) and combination thereof, from the precursor ion (black) or primary fragments α^+ (green), w^+ (blue) and y^+ (red).

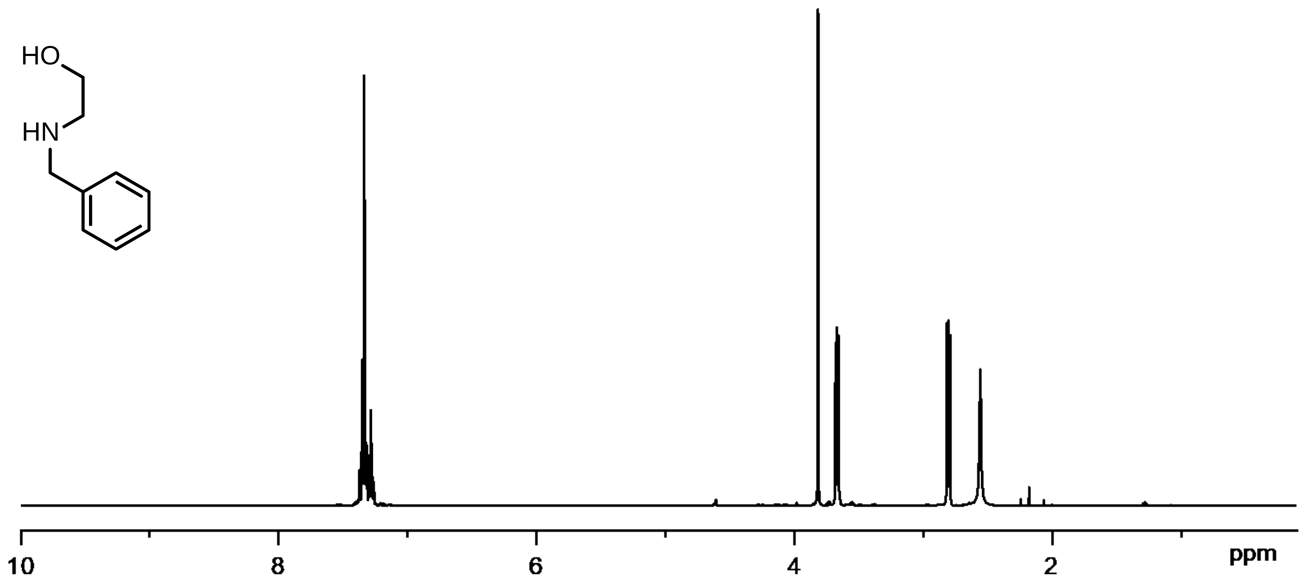

Figure S16. ^{1}H NMR spectra of **a**.

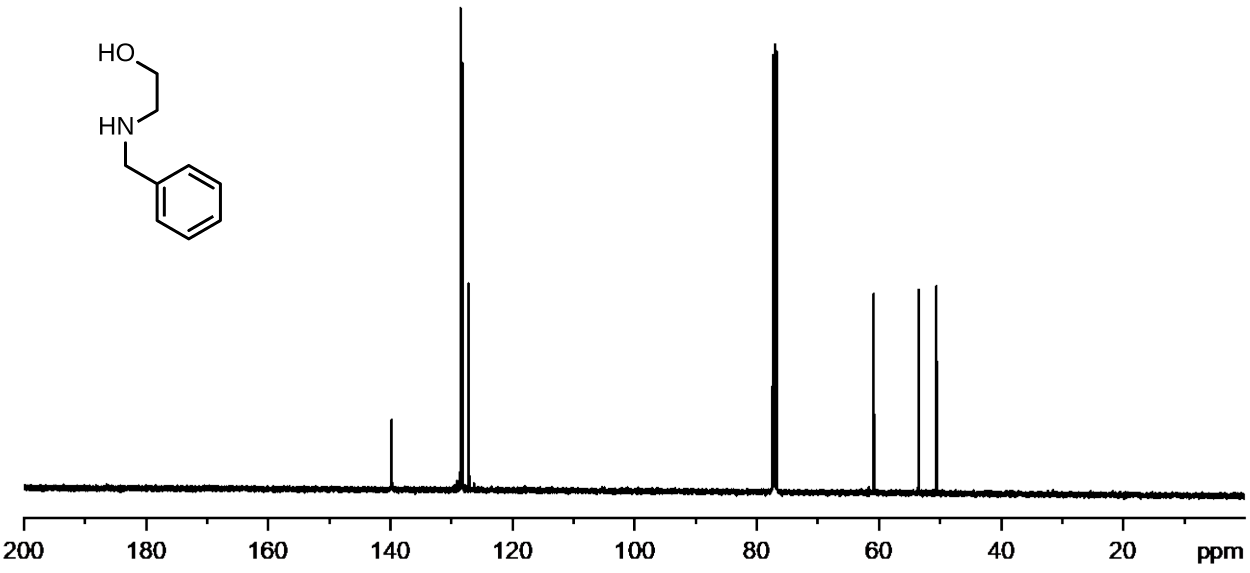

Figure S17. ^{13}C NMR spectra of **a**.

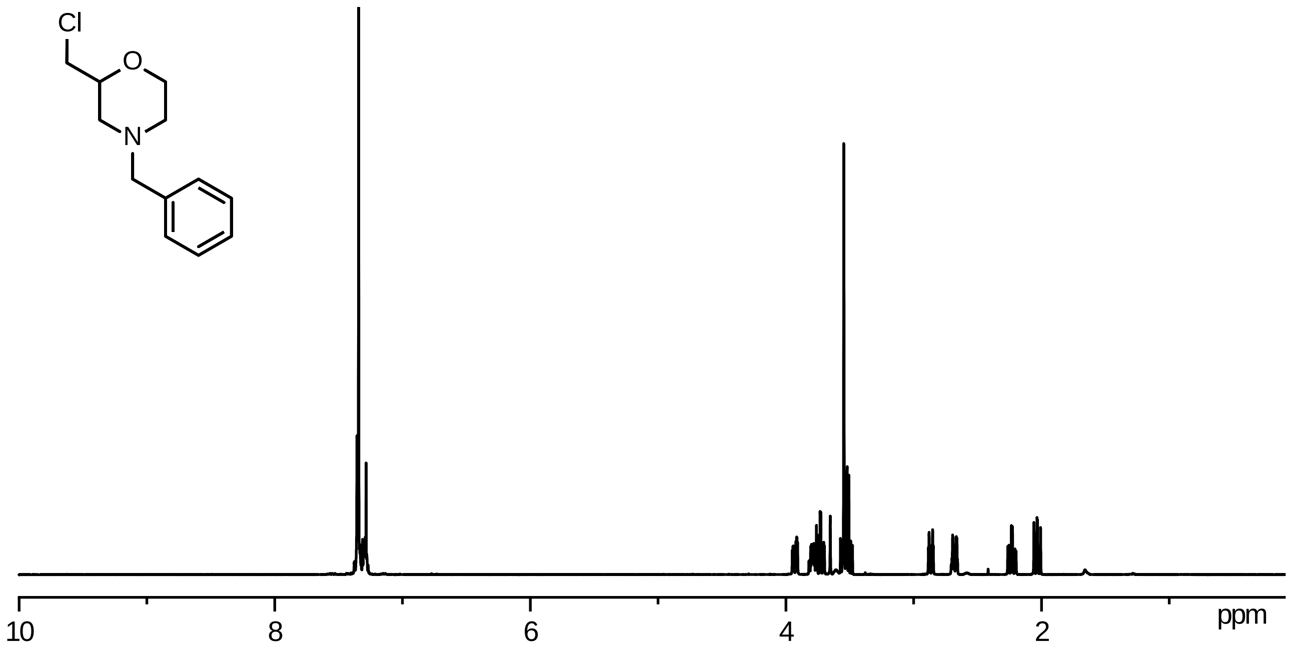

Figure S18. ^{1}H NMR spectra of **b**.

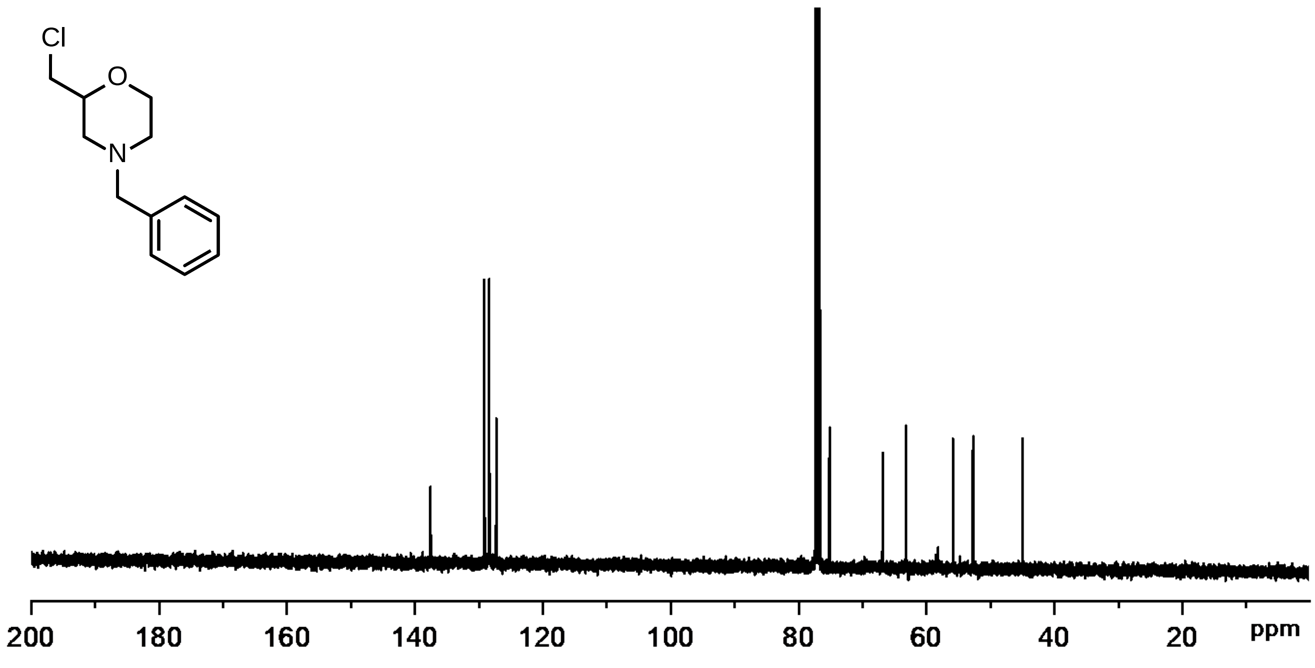

Figure S19. ^{13}C NMR spectra of **b**.

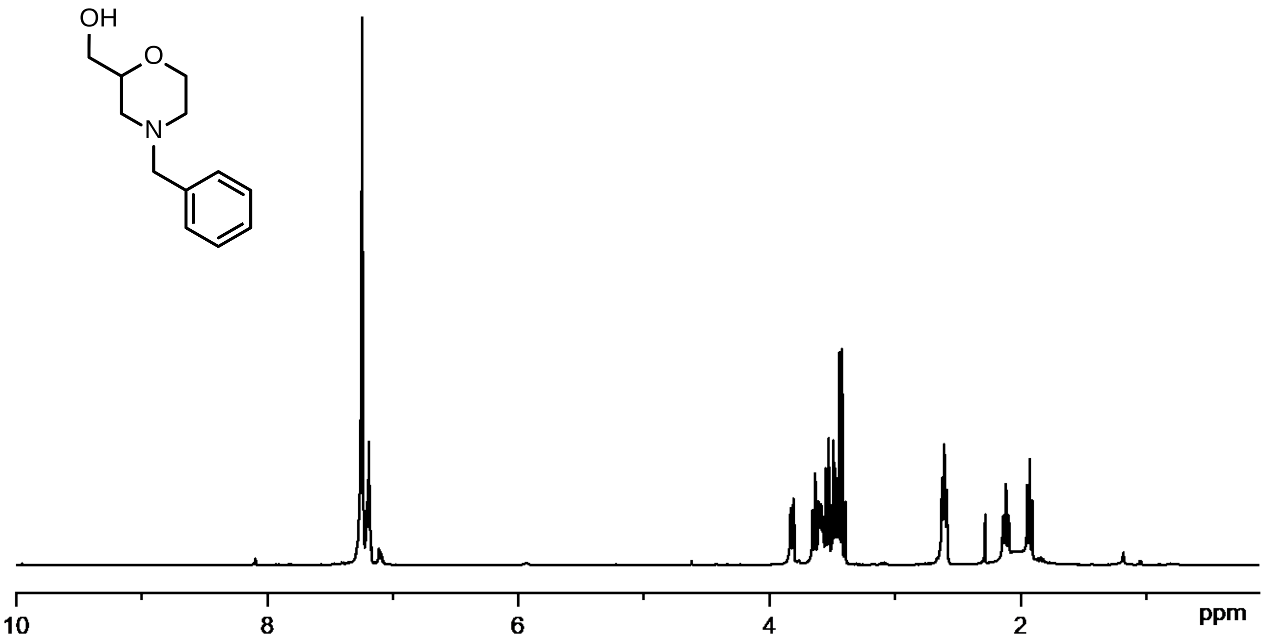

Figure S20. ^{1}H NMR spectra of **c**.

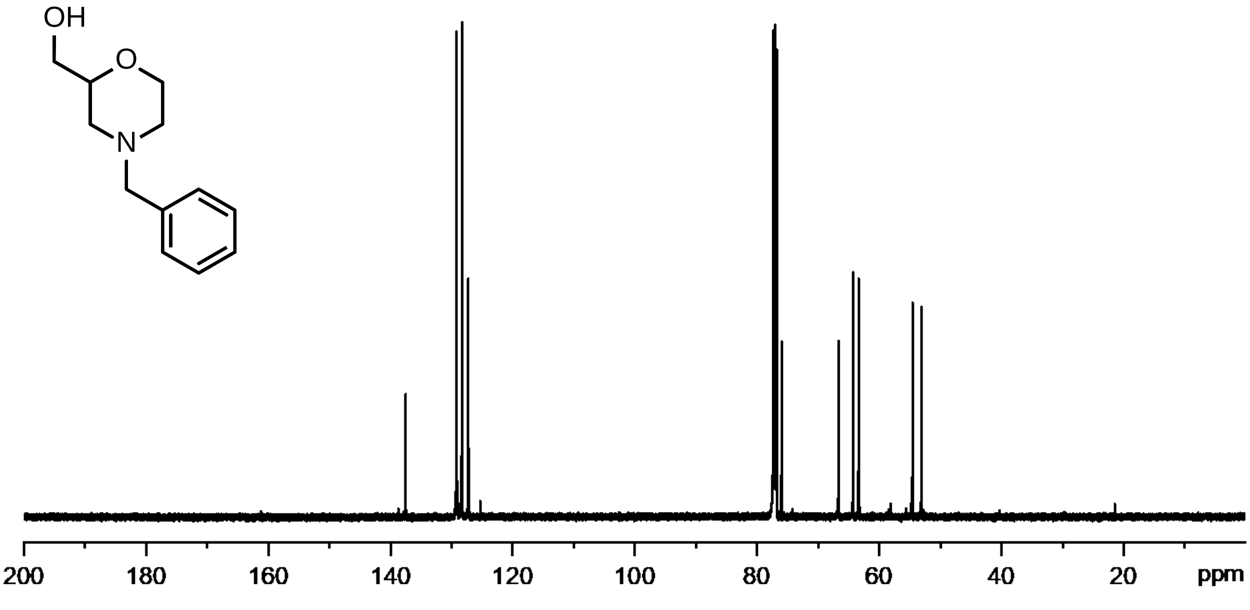

Figure S21. ^{13}C NMR spectra of **c**.

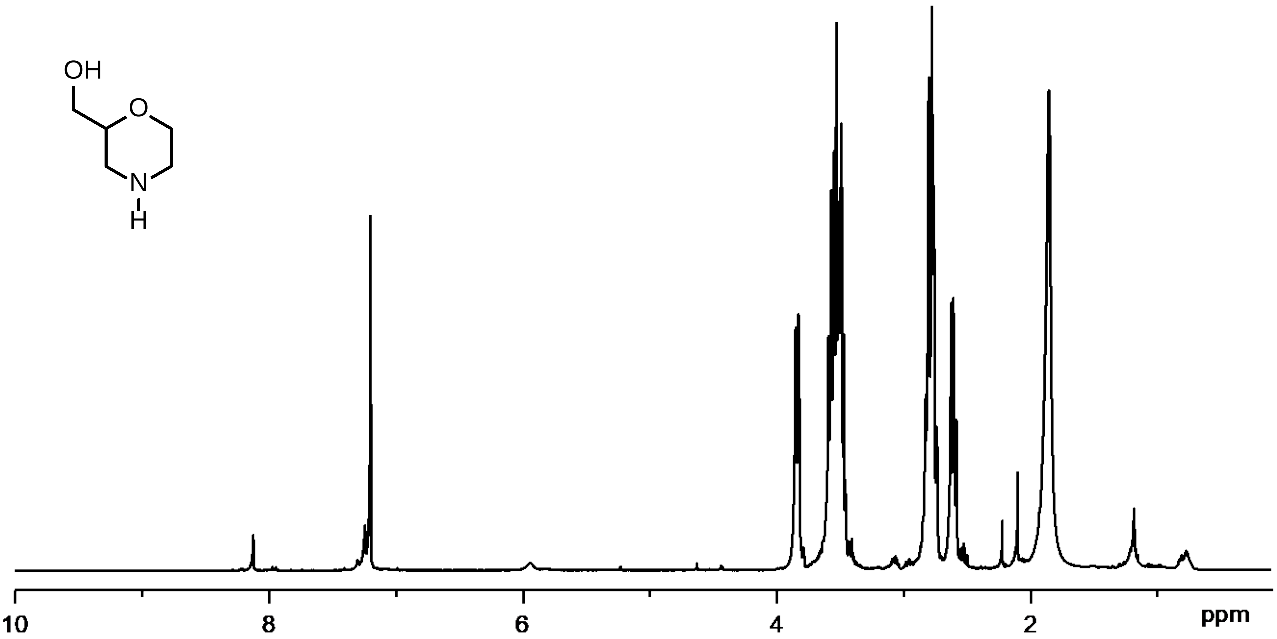

Figure S22. ^{1}H NMR spectra of **d**.

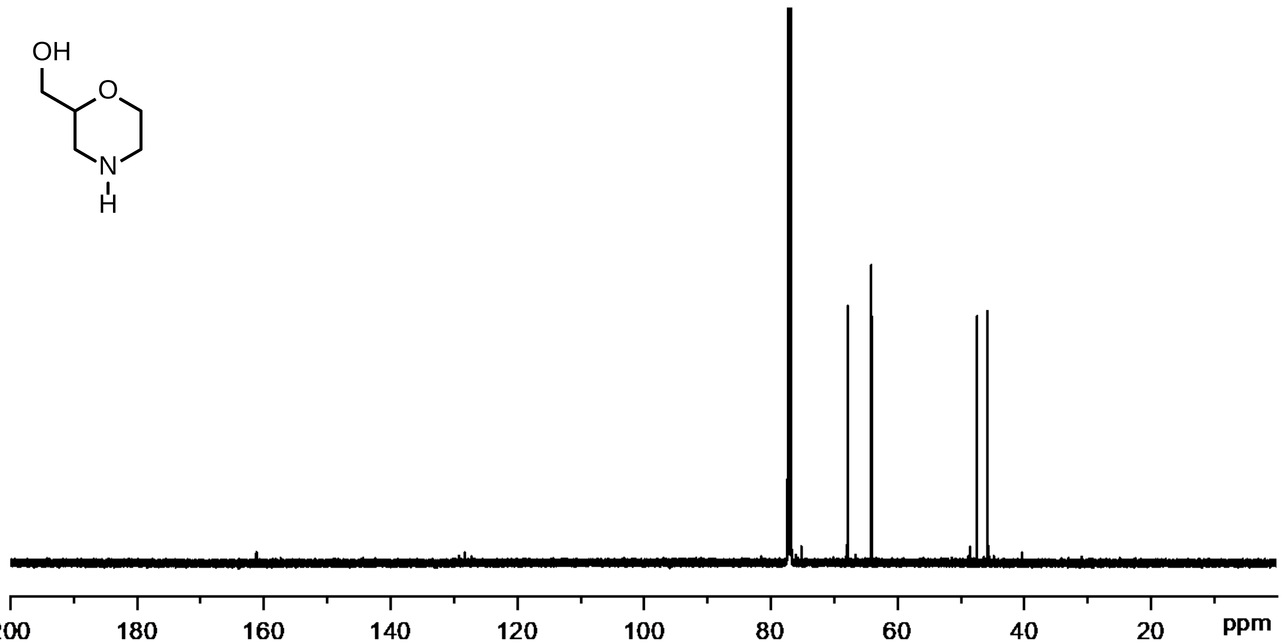

Figure S23. ^{13}C NMR spectra of **d**.

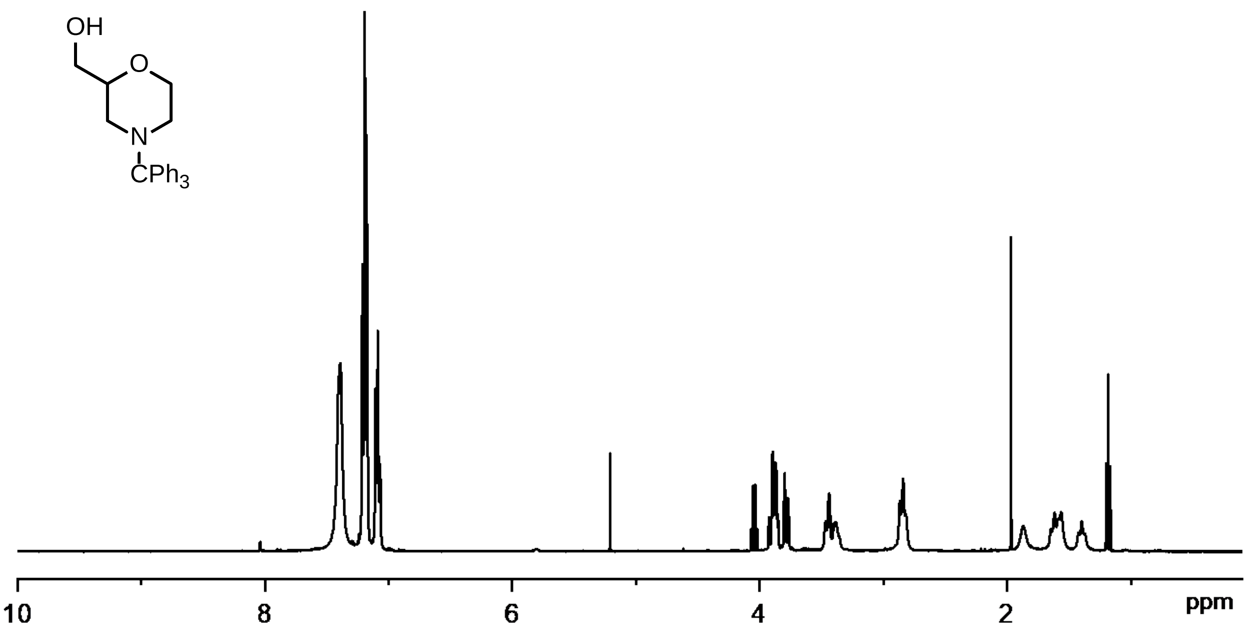

Figure S24. ^{1}H NMR spectra of **e**.

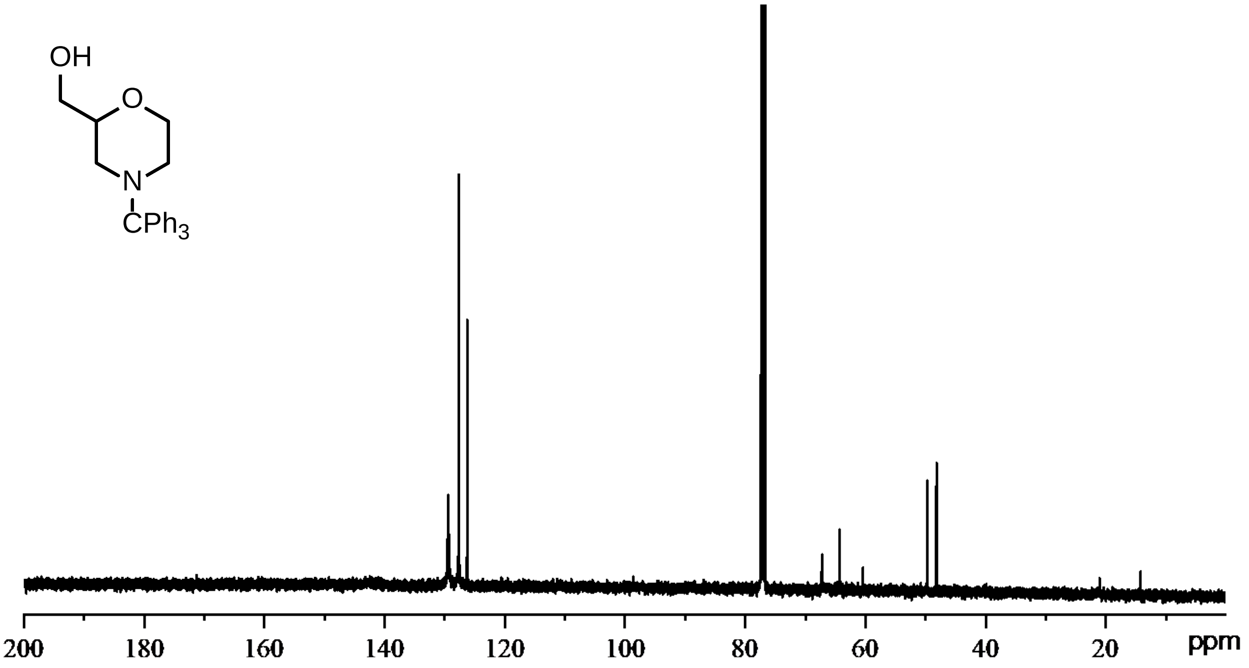

Figure S25. ^{13}C NMR spectra of **e**.

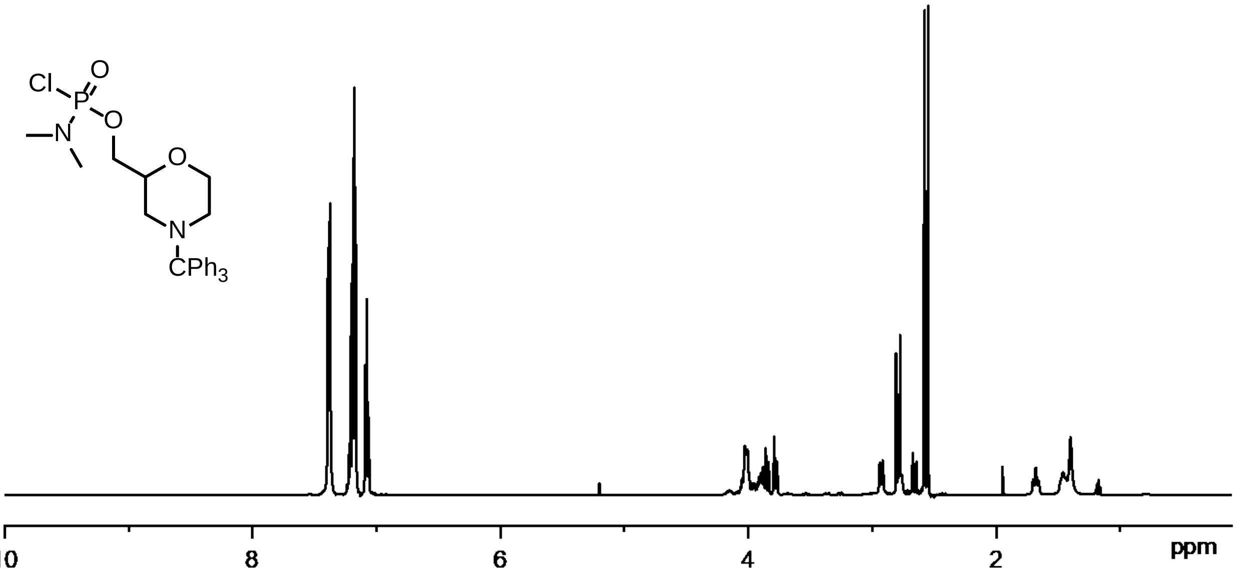

Figure S26. ^{1}H NMR spectra of **0**.

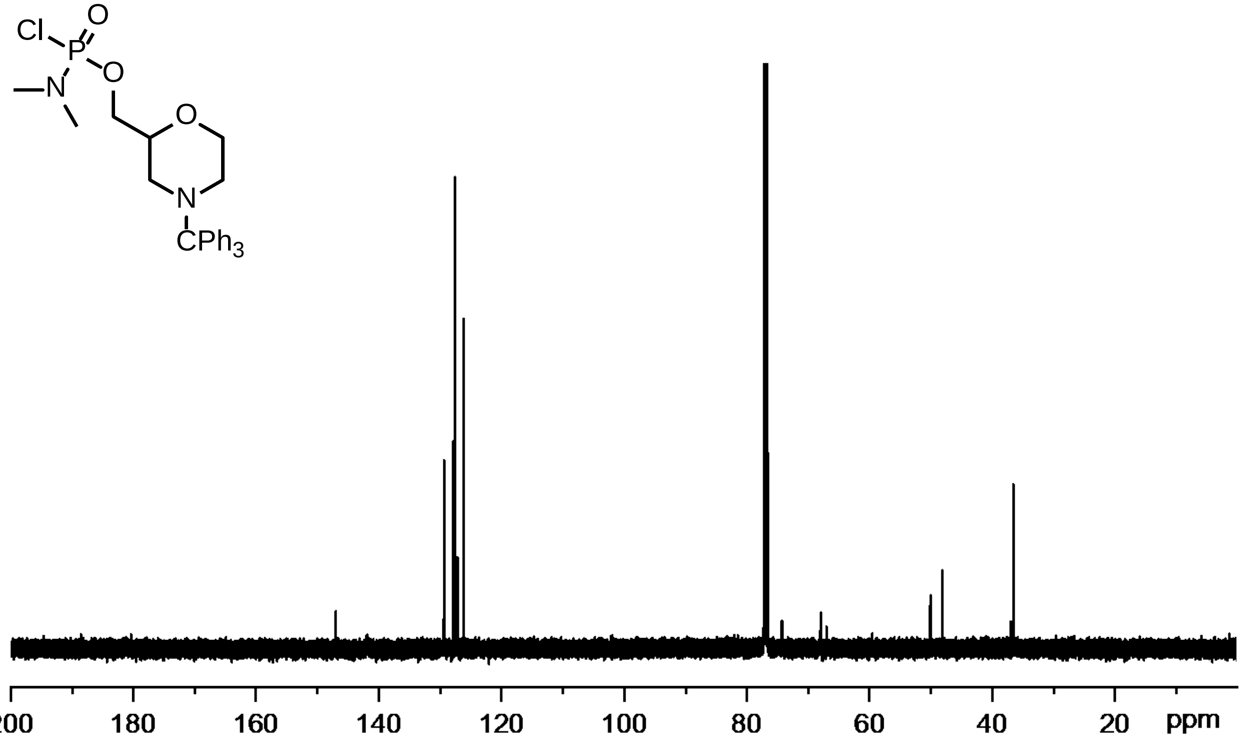

Figure S27. ^{13}C NMR spectra of **0**.

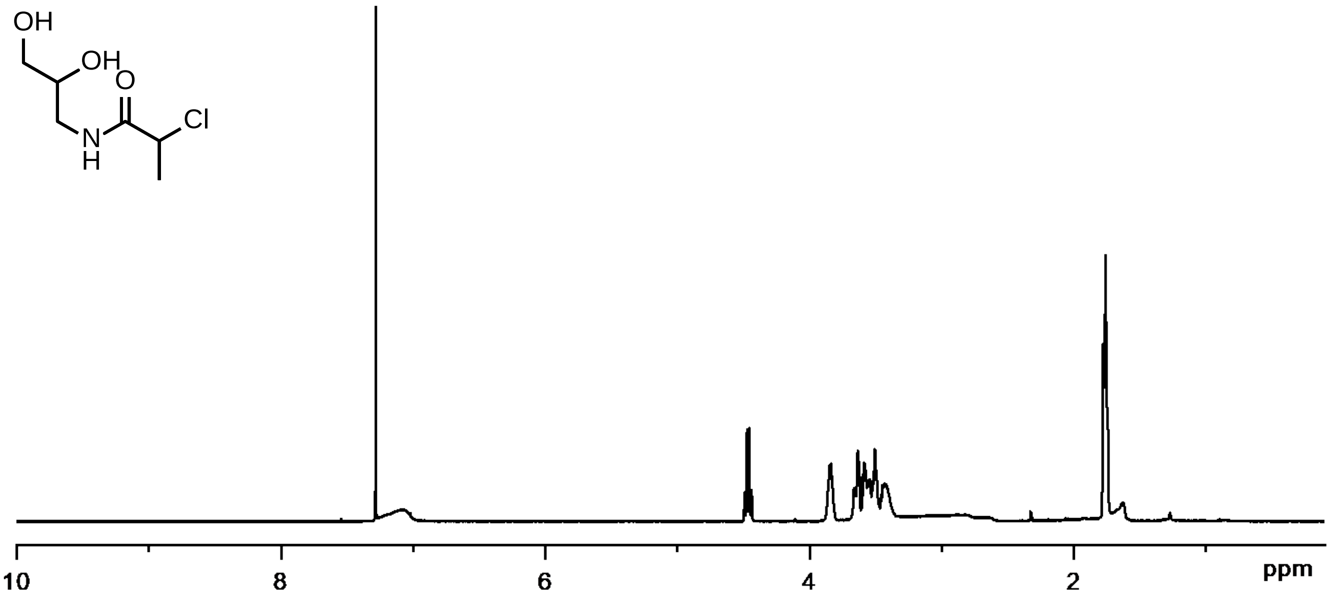

Figure S28. ^{1}H NMR spectra of **f**.

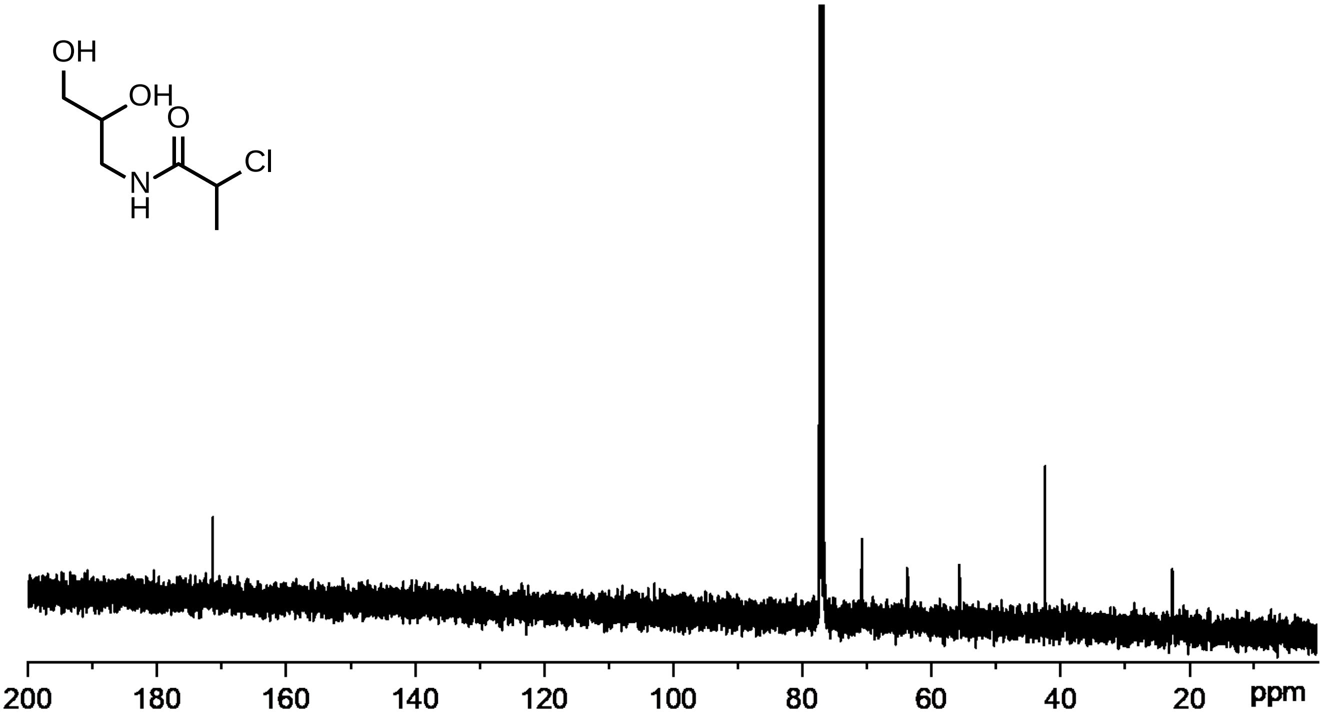

Figure S29. ^{13}C NMR spectra of **f**.

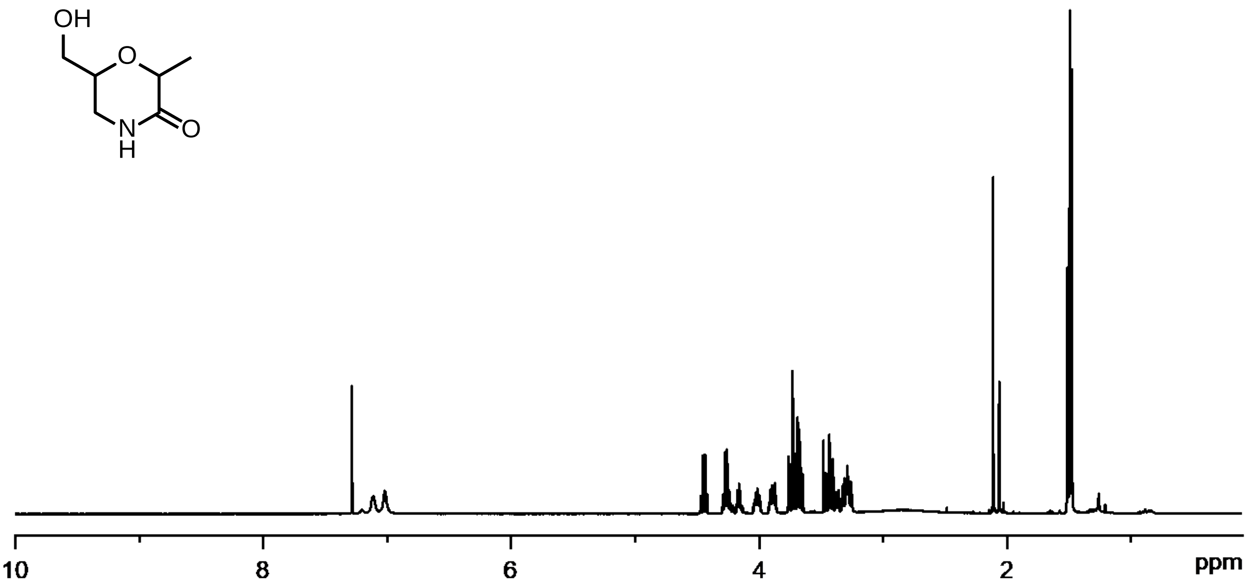

Figure S30. ^{1}H NMR spectra of **g**.

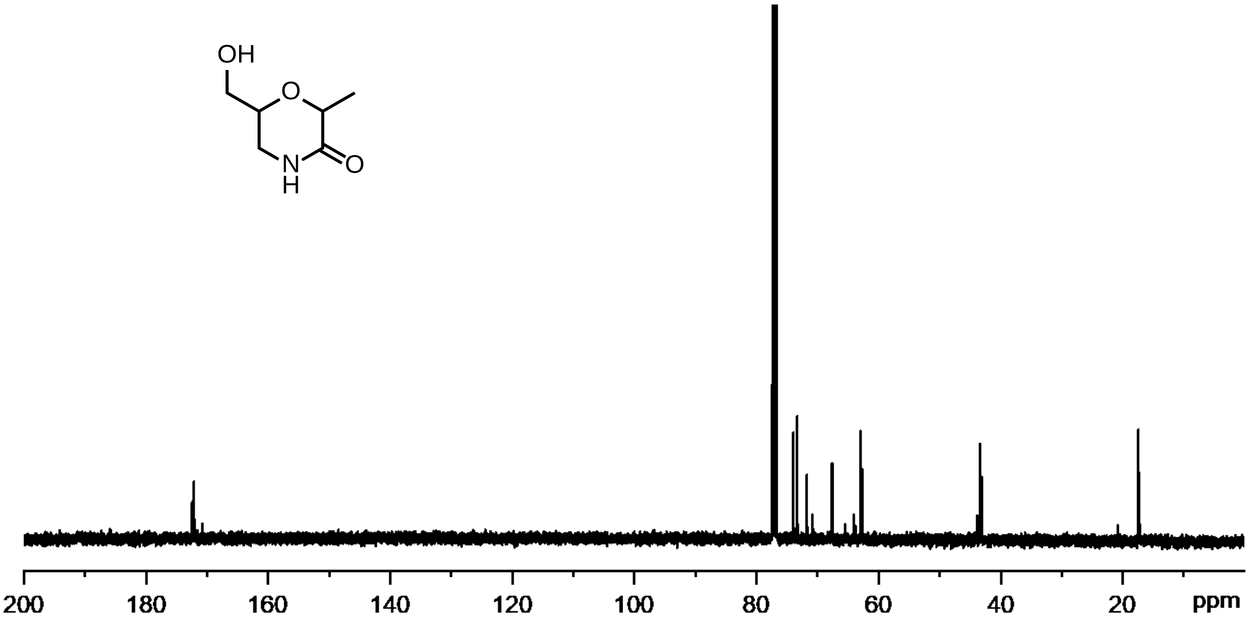

Figure S31. ^{13}C NMR spectra of **g**.

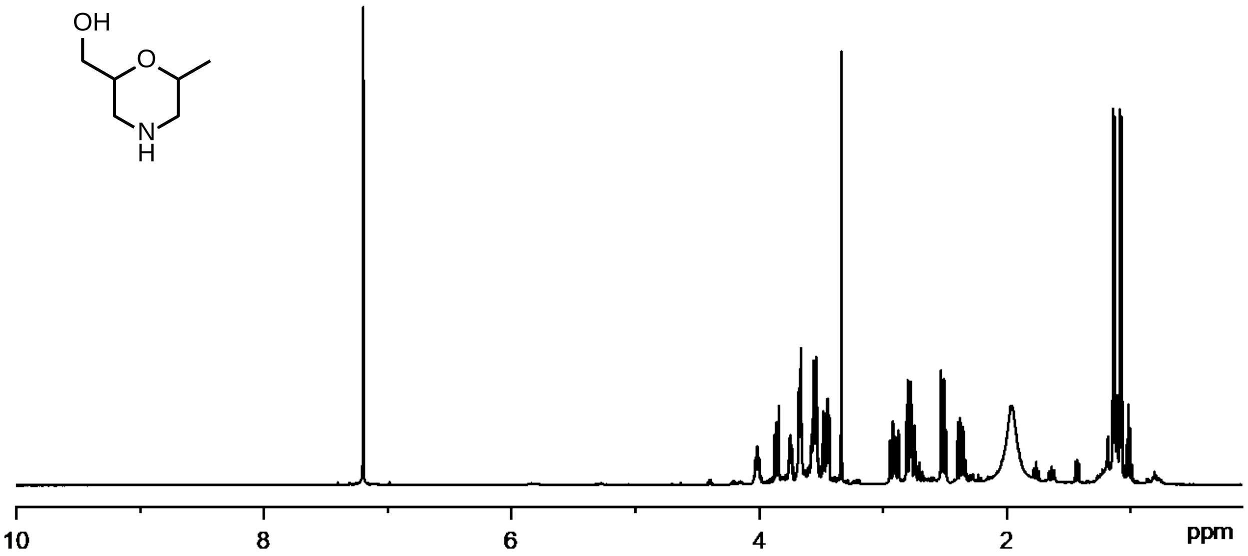

Figure S32. ^{1}H NMR spectra of **h**.

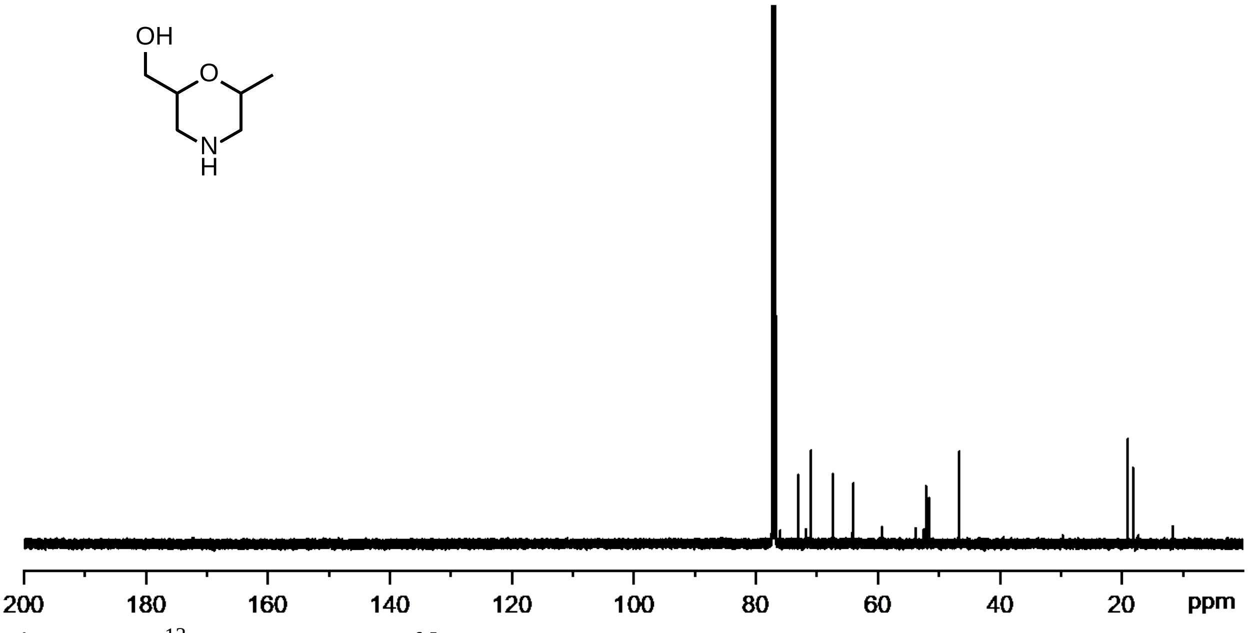

Figure S33. ^{13}C NMR spectra of **h**.

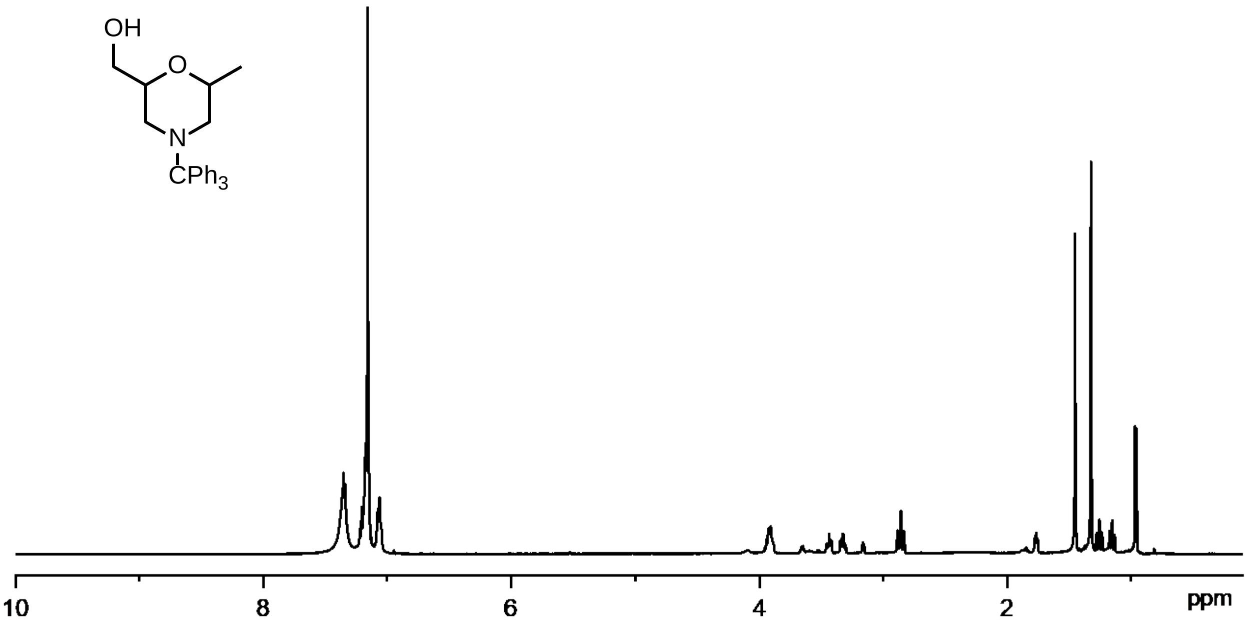

Figure S34. ^{1}H NMR spectra of **i**.

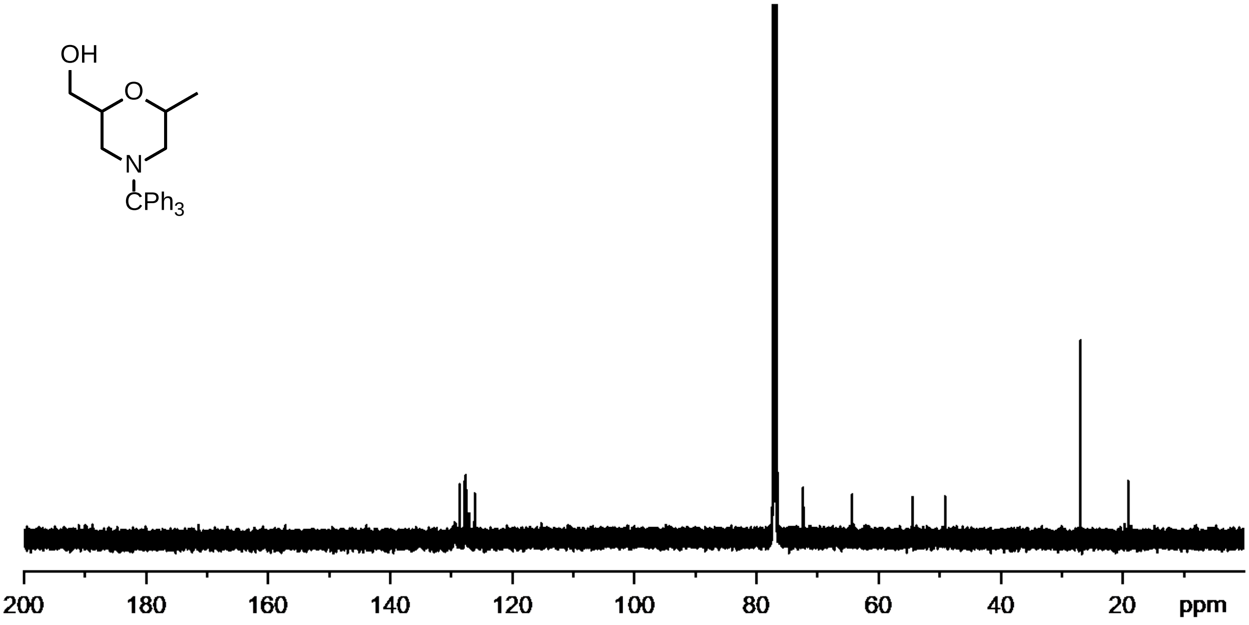

Figure S35. ^{13}C NMR spectra of **i**.

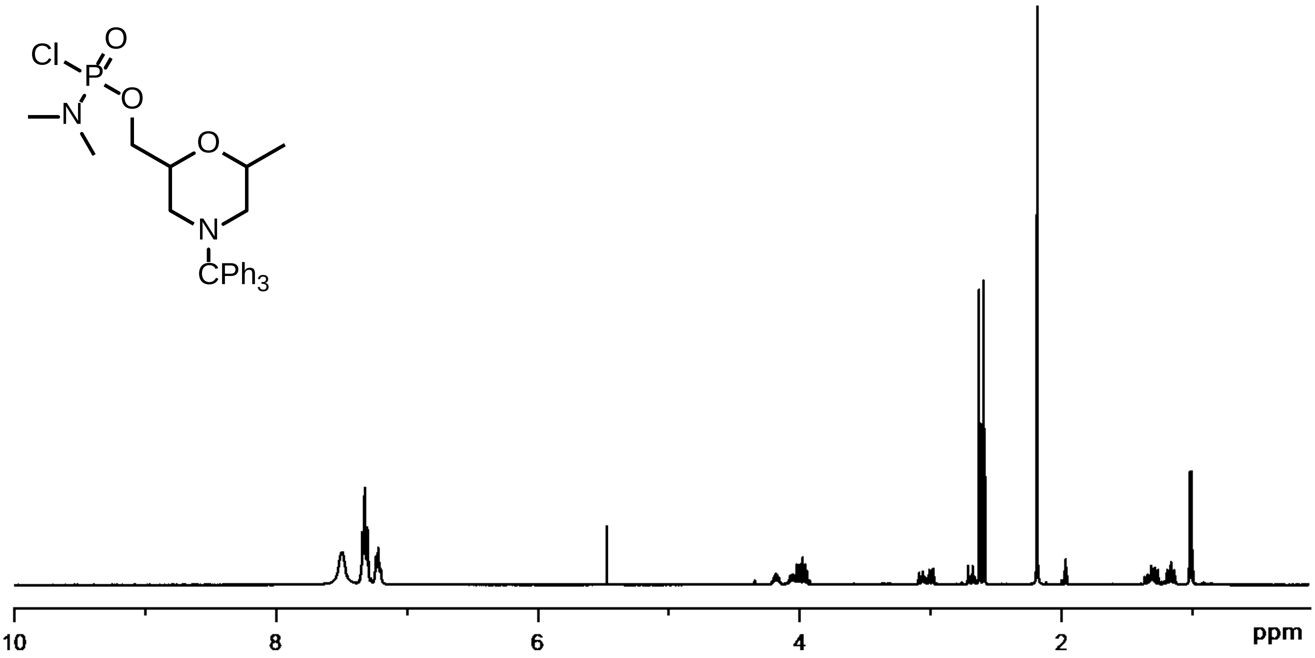

Figure S36. ^{1}H NMR spectra of **1**.

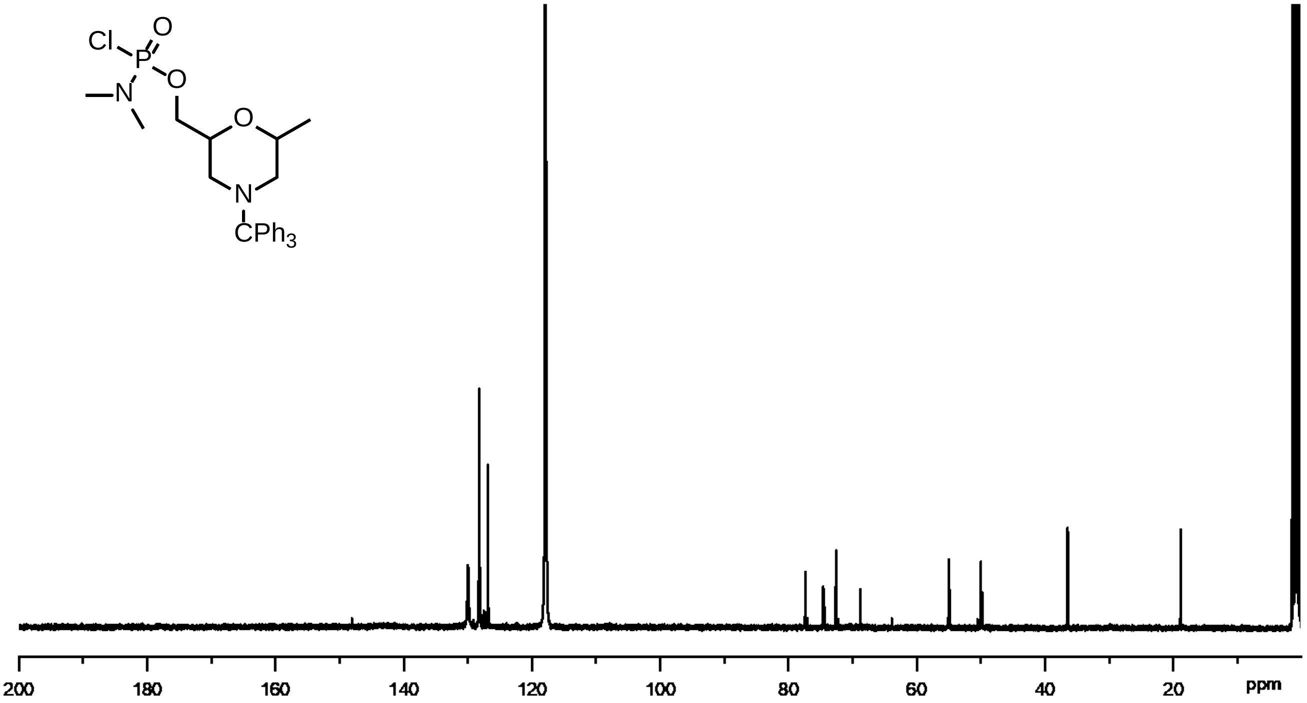

Figure S37. ^{13}C NMR spectra of **1**.

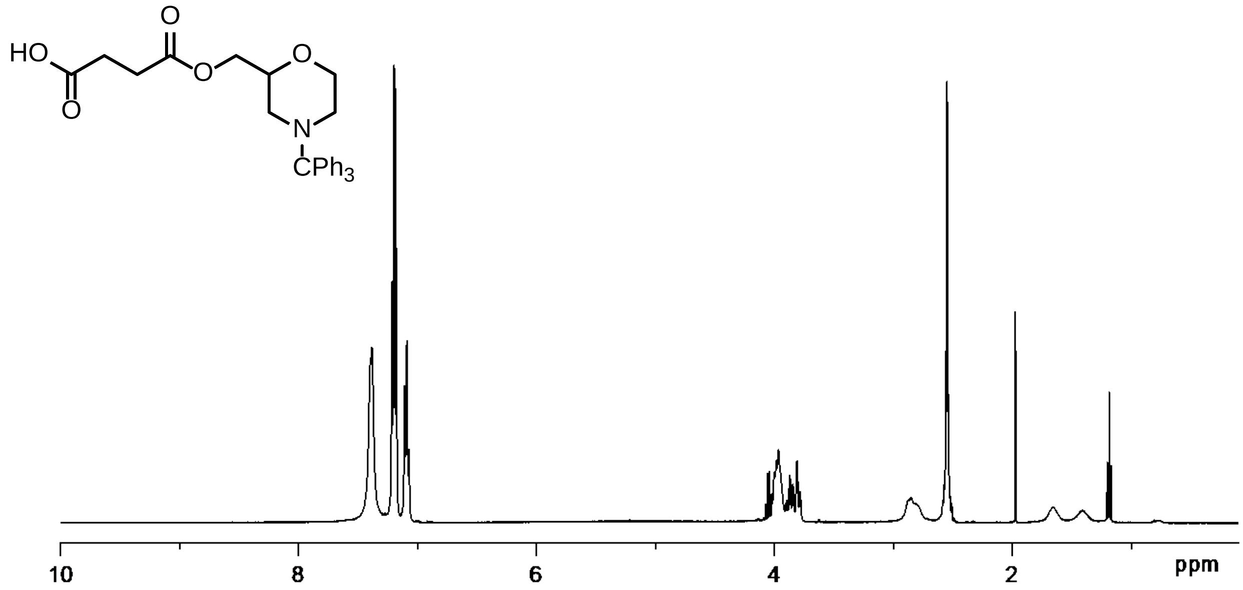

Figure S38. ^{1}H NMR spectra of **j**.

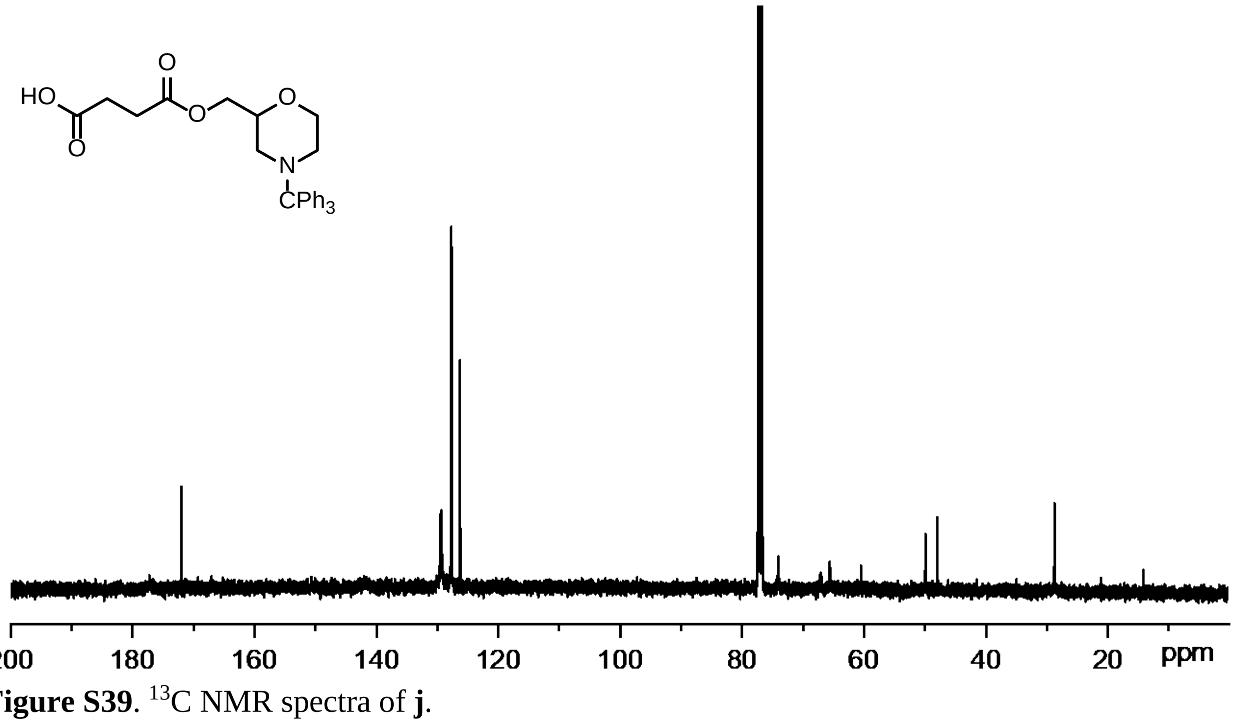

Figure S39. ^{13}C NMR spectra of **j**.

D. References.

[1] C. Gentzsch, M. Hoffmann, Y. Ohshima, N. Nose, X. Chen, T. Higuchi, M. Decker, *ChemMedChem* **2021**, *16*, 1427-1437.

[2] S. Berg, L.-G. Larsson, L. Rényi, S. B. Ross, S.-O. Thorberg, G. Thorell-Svantesson, *J. Med. Chem.* **1998**, *41*, 1934-1942.

[3] E. Brenner, R. M. Baldwin, G. Tamagnan, *Org. Lett.* **2005**, *7*, 937-939.
